# Comparison of Group-Level and Individualized Brain Regions for Measuring Change in Longitudinal Tau Positron Emission Tomography in Alzheimer Disease

**DOI:** 10.1001/jamaneurol.2023.1067

**Published:** 2023-05-08

**Authors:** Antoine Leuzy, Alexa Pichet Binette, Jacob W. Vogel, Gregory Klein, Edilio Borroni, Matteo Tonietto, Olof Strandberg, Niklas Mattsson-Carlgren, Sebastian Palmqvist, Michael J. Pontecorvo, Leonardo Iaccarino, Erik Stomrud, Rik Ossenkoppele, Ruben Smith, Oskar Hansson

**Affiliations:** 1 Clinical Memory Research Unit, Department of Clinical Sciences, Lund University, Malmö, Sweden; 2 Penn/CHOP Lifespan Brain Institute, University of Pennsylvania, Philadelphia; 3 Department of Psychiatry, University of Pennsylvania, Philadelphia; 4 F. Hoffmann-La Roche Ltd, Basel, Switzerland; 5 Department of Neurology, Skåne University Hospital, Lund, Sweden; 6 Wallenberg Centre for Molecular Medicine, Lund University, Lund, Sweden; 7 Memory Clinic, Skåne University Hospital, Lund, Sweden; 8 Avid Radiopharmaceuticals, Philadelphia, Pennsylvania; 9 Eli Lilly and Company, Indianapolis, Indiana; 10 Alzheimer Center Amsterdam, Neurology, Vrije Universiteit Amsterdam, Amsterdam UMC location VUmc, Amsterdam, the Netherlands; 11 Amsterdam Neuroscience, Neurodegeneration, Amsterdam, the Netherlands

## Abstract

**Importance:**

Longitudinal tau positron emission tomography (PET) is a relevant outcome in clinical trials evaluating disease-modifying therapies in Alzheimer disease (AD). A key unanswered question is whether the use of participant-specific (individualized) regions of interest (ROIs) is superior to conventional approaches where the same ROI (group-level) is used for each participant.

**Objective:**

To compare group- and participant-level ROIs in participants at different stages of the AD clinical continuum in terms of annual percentage change in tau-PET standardized uptake value ratio (SUVR) and sample size requirements.

**Design, Setting, and Participants:**

This was a longitudinal cohort study with consecutive participant enrollment between September 18, 2017, and November 15, 2021. Included in the analysis were participants with mild cognitive impairment and AD dementia from the prospective and longitudinal Swedish Biomarkers For Identifying Neurodegenerative Disorders Early and Reliably 2 (BioFINDER-2) study; in addition, a validation sample (the AVID 05e, Expedition-3, Alzheimer’s Disease Neuroimaging Initiative [ADNI], and BioFINDER-1 study cohorts) was also included.

**Exposures:**

Tau PET (BioFINDER-2, [^18^F]RO948; validation sample, [^18^F]flortaucipir), 7 group-level (5 data-driven stages, meta-temporal, whole brain), and 5 individualized ROIs.

**Main Outcomes and Measures:**

Annual percentage change in tau-PET SUVR across ROIs. Sample size requirements in simulated clinical trials using tau PET as an outcome were also calculated.

**Results:**

A total of 215 participants (mean [SD] age, 71.4 (7.5) years; 111 male [51.6%]) from the BioFINDER-2 study were included in this analysis: 97 amyloid-β (Aβ)–positive cognitively unimpaired (CU) individuals, 77 with Aβ-positive mild cognitive impairment (MCI), and 41 with AD dementia. In the validation sample were 137 Aβ-positive CU participants, 144 with Aβ-positive MCI, and 125 with AD dementia. Mean (SD) follow-up time was 1.8 (0.3) years. Using group-level ROIs, the largest annual percentage increase in tau-PET SUVR in Aβ-positive CU individuals was seen in a composite ROI combining the entorhinal cortex, hippocampus, and amygdala (4.29%; 95% CI, 3.42%-5.16%). In individuals with Aβ-positive MCI, the greatest change was seen in the temporal cortical regions (5.82%; 95% CI, 4.67%-6.97%), whereas in those with AD dementia, the greatest change was seen in the parietal regions (5.22%; 95% CI, 3.95%-6.49%). Significantly higher estimates of annual percentage change were found using several of the participant-specific ROIs. Importantly, the simplest participant-specific approach, where change in tau PET was calculated in an ROI that best matched the participant’s data-driven disease stage, performed best in all 3 subgroups. For the power analysis, sample size reductions for the participant-specific ROIs ranged from 15.94% (95% CI, 8.14%-23.74%) to 72.10% (95% CI, 67.10%-77.20%) compared with the best-performing group-level ROIs. Findings were replicated using [^18^F]flortaucipir.

**Conclusions and Relevance:**

Finding suggest that certain individualized ROIs carry an advantage over group-level ROIs for assessing longitudinal tau changes and increase the power to detect treatment effects in AD clinical trials using longitudinal tau PET as an outcome.

## Key Points

**Question:**

Does defining regions of interest (ROIs) for each participant (individualized) improve the sensitivity to tau accumulation compared with the use of a conventional approach where each participant is assigned the same ROI (group-level)?

**Findings:**

Significantly higher estimates of annual change in tau positron emission tomography (PET) were found using several of the participant-specific ROIs. Importantly, the simplest individualized approach, where change in tau PET was calculated in an ROI that best matched the participant’s data-driven disease stage, performed best.

**Meaning:**

Individualized ROIs carry an advantage over group-level ROIs for assessing longitudinal tau PET and can increase the sensitivity to detect treatment effects in AD trials.

## Teaser

This cohort study investigates if defining participant-level regions of interest improves sensitivity to tau accumulation on positron emission tomography compared with group-level regions of interest.

## Introduction

The neuropathological hallmarks of Alzheimer disease (AD) include deposition of extracellular amyloid β (Aβ) and intracellular hyperphosphorylated tau. In contrast to Aβ pathology, which occurs in cortical regions decades before dementia onset, tau pathology is thought to emerge in circumscribed regions of the medial temporal lobe in early adulthood, before spreading into cortical regions around the time of symptom onset.^1^ Studies using positron emission tomography (PET) ligands with high affinity for the tau aggregates formed in AD have shown that the degree and topography of cortical tau-PET retention overlap strongly with neurodegeneration^2^ and associate with cognitive decline.^2,3^ As such, tau accumulation is a relevant intervention target and potential outcome measure in AD.^4^

The spread of tau in AD has classically been thought to follow a stereotypical spatiotemporal pattern based on postmortem studies^5,6^—from the (trans)entorhinal cortex into the hippocampus and inferior temporal lobe, before reaching cortical association areas (Braak staging scheme). However, autopsy^7^ and both cross-sectional^8,9,10^ and longitudinal^11,12,13,14,15^ tau-PET studies have found substantial interindividual differences in the deposition and accumulation of tau, with important deviations from the traditional Braak model.^7,8,9,10^ Indeed, several distinct subtypes of tau pathology were recently identified using tau PET, with these exhibiting distinct demographic and cognitive profiles, as well as differing longitudinal outcomes.^16^ Although the mechanisms underlying this variability remain, as yet, unclear, they may be associated with individual differences in brain organization (eg, variation in axonal connectivity patterns), regional vulnerability (eg, deposition of tau along different networks), or variation in disease biochemistry (eg, individual differences in enzymatic activity, copathology, or other biological processes).^17^

Longitudinal tau PET is increasingly used as an outcome measure to detect either drug target engagement or efficacy in AD clinical trials evaluating disease-modifying therapies.^18^ Recent work comparing tau PET and cognition as outcomes in clinical trials showed that significantly fewer participants were required to detect a meaningful change in the rate of tau accumulation compared with the rate of cognitive decline.^19^ However, interindividual heterogeneity in the pattern of tau spread poses a challenge to the accurate prediction of tau progression at the individual level.^11,12,13,14,15^ The use of participant-specific (individualized) approaches for the prediction of future tau spreading may help increase the sensitivity to detect treatment effects and help reduce the number of patients included into these trials. Preliminary findings using one such approach showed that defining regions of interest (ROIs) for each participant improved the sensitivity to tau accumulation and significantly reduced required sample sizes when compared with the use of conventional (eg, Braak stages, temporal and whole-brain meta-ROIs) approaches where each participant is assigned the same (group-level) ROI.^20^ In the present study, we aimed to expand on work on participant-specific ROIs by comparing group-level and individualized ROIs defined using a range of methods differing in complexity level across several metrics using longitudinal [^18^F]RO948 tau PET in participants at different stages of the AD clinical continuum. The results were validated in an independent data set with longitudinal [^18^F]flortaucipir.

## Methods

### Participants

This cohort study received ethical approval from the Regional Ethical Committee in Lund, Sweden. Approval for PET imaging was obtained from the Swedish Medicines and Products Agency and the local Radiation Safety Committee at Skåne University Hospital in Sweden. All participants gave written informed consent. We included cognitively unimpaired (CU) individuals, patients with mild cognitive impairment (MCI), and those with AD dementia from the prospective and longitudinal Swedish Biomarkers For Identifying Neurodegenerative Disorders Early and Reliably 2 (BioFINDER-2) study.^21^ Participants were enrolled between September 18, 2017, and November 15, 2021. Inclusion and exclusion criteria have been described elsewhere (eMethods 1 in Supplement 1).^22,23^ CU individuals were 60 years or older and did not have MCI or dementia.^22,23^ Exclusion criteria included presence of objective cognitive impairment, severe somatic disease, and current alcohol/substance misuse. Patients with MCI fulfilled the *Diagnostic and Statistical Manual of Mental Disorders* (Fifth Edition) criteria for mild neurocognitive disorder whereas patients with AD dementia fulfilled the *DSM-5* criteria for major cognitive impairment due to AD.^24^ Aβ status was defined using CSFAβ42/Aβ40, as previously described.^23^ This study followed the Strengthening the Reporting of Observational Studies in Epidemiology (STROBE) reporting guidelines.

### Image Acquisition and Processing

[^18^F]RO948 PET was performed on digital scanners (Discovery MI [GE Healthcare]) 70 to 90 minutes after injection, as described previously.^22^ As some off-target binding has been described in the meninges for [^18^F]RO948,^22^ no smoothing was performed during image reconstruction. To further reduce the possibility an off-target signal, a more accurate meningeal segmentation was obtained using T1/T2 magnetic resonance imaging (with Sequence Adaptive Multimodal Segmentation [SAMSEG] FreeSurfer utility [Laboratory for Computational Neuroimaging]),^25^ which was then used to prune the adjacent FreeSurfer ROIs (eMethods 2 in Supplement 1). The corrected ROI set was then used in a geometric transfer matrix (GTM) partial volume error correction.^26^ Serial high-resolution T1-weighted magnetic resonance images were acquired (3T MAGNETOM Prisma [Siemens Healthineers]) for PET image coregistration, template normalization, and segmentation using longitudinal FreeSurfer, version 6.0.^27^ Standardized uptake value ratio (SUVR) images were created using the inferior cerebellar cortex as the reference region. A sensitivity analysis was performed using an alternative reference region consisting of the whole cerebellum, brainstem, and eroded subcortical white matter. In keeping with the ROI-based analyses that were corrected for partial volume effects using GTM, voxelwise analyses were performed using region-based voxelwise correction,^28^ a partial-volume technique that extends the GTM method and performs a voxelwise correction of the entire image.

### Tau-PET ROI Definition

**Figure 1.**
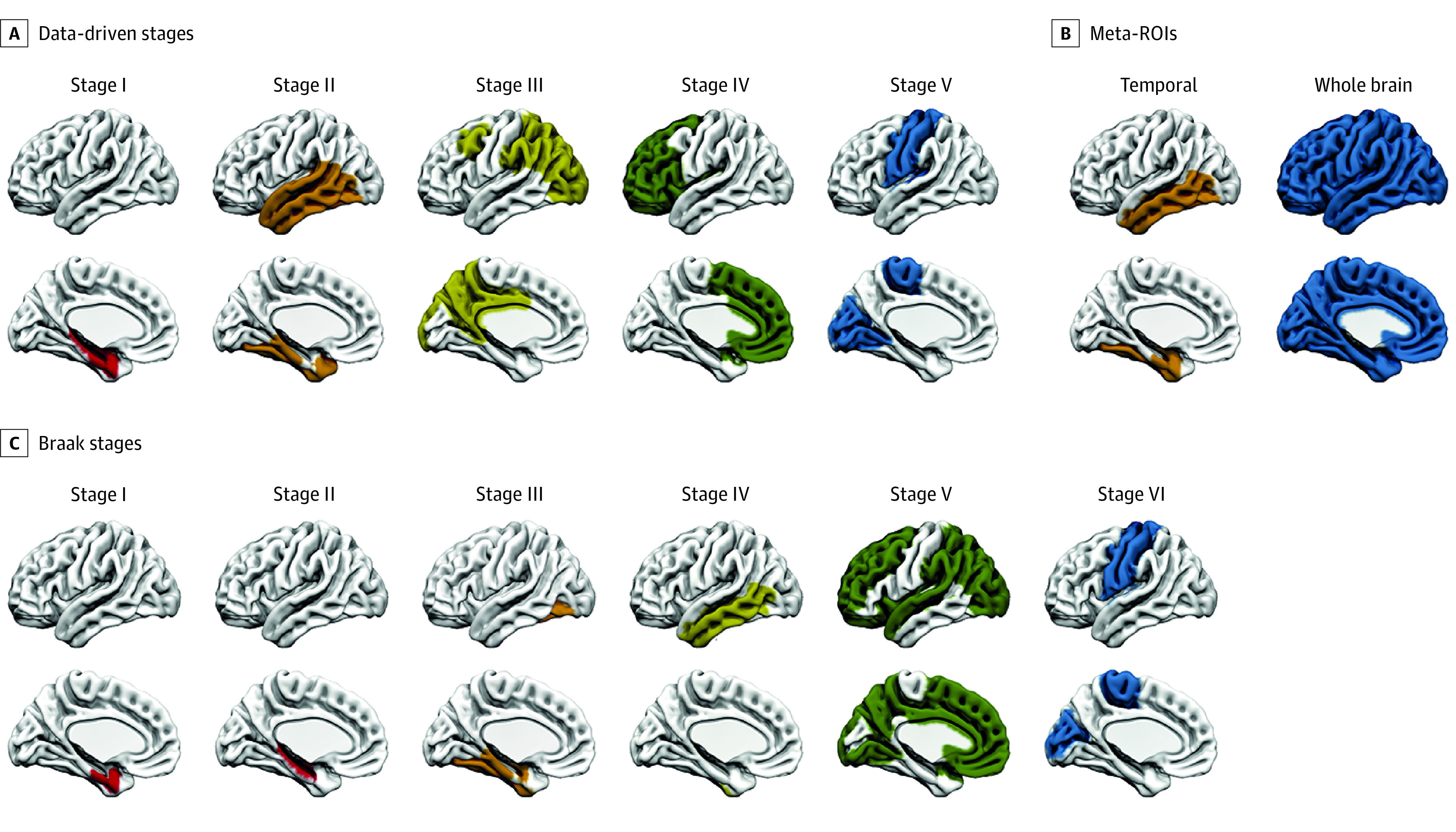
Group-Level Regions of Interest (ROIs) Group-level ROIs include previously published data-driven stages for [^18^F]RO948 (A), temporal and whole-brain meta-ROIs (B), and Braak stages (C).

Complete details for group-level and individualized ROIs are included in eMethods 3 and eTables 1, 2, and 3 in Supplement 1. All approaches were implemented in native space. Group-level ROIs (Figure 1) included previously described stages for [^18^F]RO948,^29^ established using a data-driven approach combining clustering and event-based modeling.^30,31^ This approach identified target ROIs that were broadly consistent with widely used [^18^F]flortaucipir-derived Braaklike imaging stages and covered the full spectrum of AD tau aggregation, from early to later affected areas. In addition, we included whole-brain and temporal meta-ROIs.^32^ A sensitivity analysis was performed using Braak ROIs.^33^

**Figure 2.**
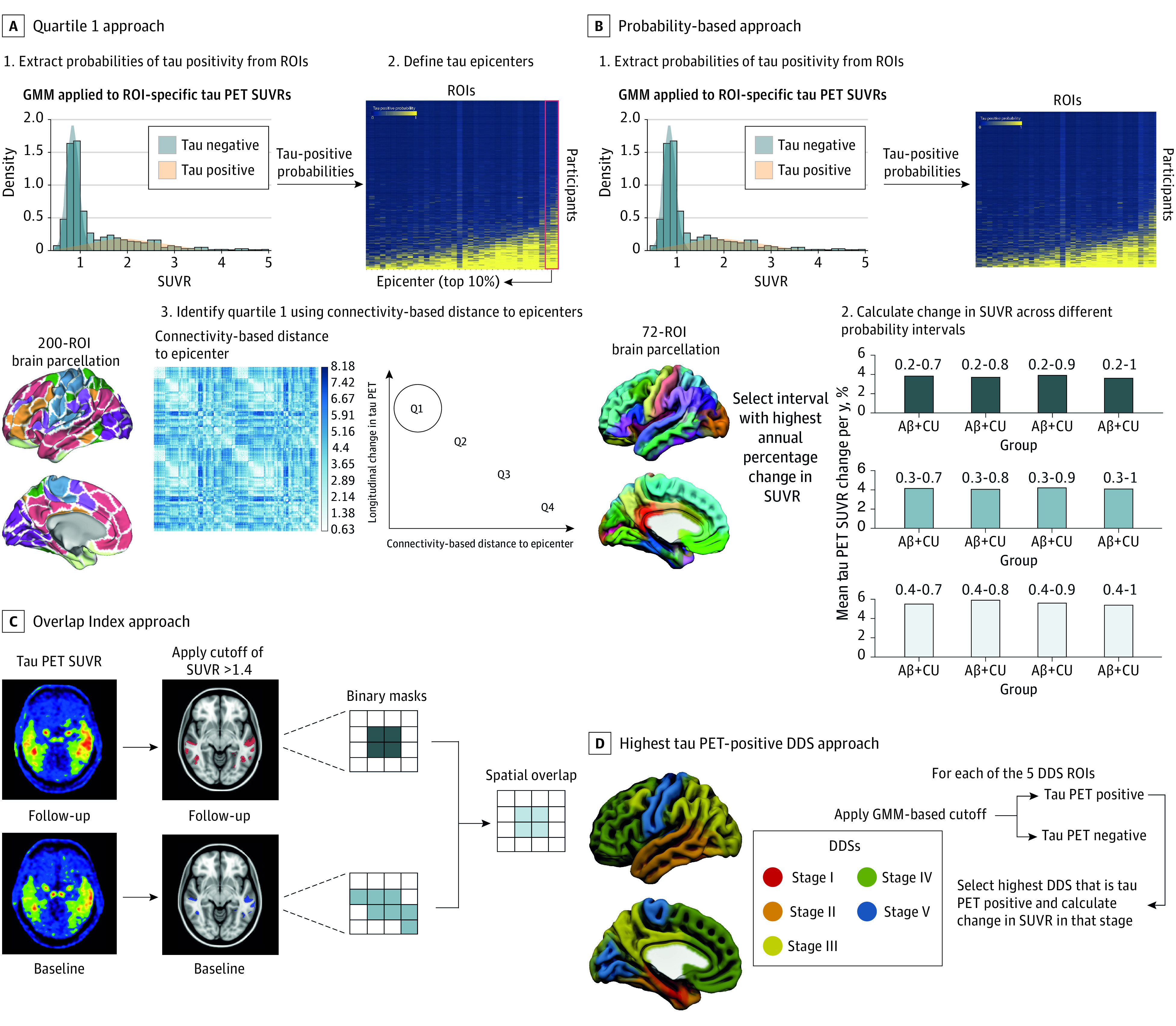
Approaches to Derive Individualized Regions of Interest (ROIs) A, In the quartile 1 approach, we extracted gaussian mixture modeling (GMM)–based probabilities of being tau positive from all brain regions in the Schaefer brain atlas and defined the top 10% of ROIs as tau epicenters (ie, brain regions in which tau emerges first). After calculating the connectivity-based distance of each ROI to the participant-specific epicenters, ROIs were grouped into nonoverlapping quartiles on the basis of their connectivity-based distance to the epicenter (ie, quartile 1 [Q1] is closest to the epicenter). B, In the probability-based approach, GMM-based probabilities of being tau positive were first extracted from the FreeSurfer (Laboratory for Computational Neuroimaging) atlas and change in tau–positron emission tomography (PET) standardized uptake value ratio (SUVR) calculated across different probability intervals. In order to limit the number of intervals, 0.2 to 0.4 (ie, 0.2, 0.3, 0.4) and 0.7 to 1 (ie, 0.7, 0.8 0.9, 1) were used as lower and upper start values, respectively. The interval that resulted in the highest annual percentage change in tau-PET SUVR across participants was then selected for use. The approach is illustrated for amyloid-β (Aβ)–positive cognitively unimpaired (CU) individuals. C, In the overlap index approach, change in tau PET was determined using the mask resulting from the spatial overlap of the baseline and follow-up scans. D, In the highest tau-PET–positive data-driven stage (DDS) approach, cutoffs were applied to baseline SUVR data in group-level data-driven ROIs, with change calculated using the highest (ie, latest in the tau progression cascade) ROI that was tau positive.

For individualized ROIs (Figure 2), the following 5 approaches were used: (1) quartile 1 (Q1), (2) probability based, (3) overlap index, (4) highest tau-PET–positive data-driven stage (DDS), and (5) subtype and stage inference (SUSTAIN). In Q1, as described elsewhere,^20^ Gaussian-mixture modeling (GMM) was first performed across all 200 cortical regions of the Schaefer brain atlas^34^ in order to extract the probability of being tau-PET positive for each region. After establishing participant-specific tau-PET epicenters (ie, the top 10% regions with the highest probability of having abnormal tau-PET SUVR values), the functional connectivity-based distance of each the remaining ROIs to the epicenter was determined and divided into nonoverlapping quartiles based each region’s connectivity to the epicenter.^20^ Q1, representing the top 25% regions with strongest functional connectivity to the epicenter, was used to calculate change in tau-PET SUVR. Functional connectivity data was obtained from 69 CU individuals from the ADNI cohort who were Aβ-negative and had low (global SUVR <1.30) tau-PET binding.^35^ Distance between epicenter and nonepicenter ROIs was determined by converting average participant-specific functional connectivity matrices to a distance-based connectivity matrix, with shorter path length between ROIs indicating stronger connectivity.^36^

Using the probability-based approach, the probability of being tau-PET positive was extracted for each region from the FreeSurfer atlas using GMM.^20^ Different probability intervals were then selected, with brain regions with probabilities within the selected interval combined into a composite ROI for each participant and used to calculated change in tau-PET SUVR. The probability interval yielding the highest average change across participants was then selected.

Overlap index is a recently proposed method that assesses the stability of voxels above a defined threshold,^37^ based on the assumption that suprathreshold voxels that remain stable over time represent a true positive signal due to tau pathology and not random variability. Only cortical gray matter was considered, with a mask extracted from the FreeSurfer segmentation. After obtaining masks for the baseline and follow-up scans using an SUVR threshold of greater than 1.40, their spatial overlap was determined, with the resulting mask then used to calculate change in tau-PET SUVR. In keeping with the ROI-based analyses, the analysis was performed in native space.

The highest tau-PET–positive DDS is based on in vivo tau-PET studies showing that tau aggregation in AD follows a hierarchical pattern in the cortex.^29,38,39^ We hypothesized that change in tau-PET SUVRs would be highest in those regions most recently affected. We therefore applied GMM-based cutoffs to baseline SUVR values for each of the 5 DDS ROIs (designated stages 1-5) (Figure 1) to determine which of these ROIs were affected by tau at baseline in each individual. Next, we measured longitudinal change in the ROI affected last in the tau accumulation cascade in each participant (eg, using the stage 4 ROI in an individual positive in stage ROIs 1, 2, 3, and 4) (eTable 4 in Supplement 1).

SUSTAIN is an unsupervised machine-learning technique that identifies population subgroups with common patterns of disease progression.^40^ Specifically, it first identifies subtypes and then reconstructs the trajectory of stages within each subtype, with a subtype and stage assigned to each participant. SUSTAIN has recently been used to identify 4 tau-PET subtypes in AD using [^18^F]flortaucipir.^16^

The 4-subtype model was applied to probabilistically assign individuals to 1 of 30 progressive stages along 1 of 4 subtype trajectories. In order to create an individualized ROI for each participant, the FreeSurfer regions included in each stage were grouped as follows: 1 to 10, 11 to 20, and 21 to 30.

### Power and Sensitivity Analysis Comparing Change in SUVR and Tau Extent

As preliminary data using [^18^F]GTP-1 has shown greater sensitivity to longitudinal change in tau PET using *tau extent*, defined as the number of voxels above a defined threshold divided by the total number of voxels in that region (ie, the percentage of abnormal voxels within a given region), in the temporal meta-ROI,^41^ we compared sample size reduction using annual percentage change in tau extent and SUVR for [^18^F]RO948 in this region (eMethods 3 in Supplement 1).

### Independent Validation Sample

Participants (Aβ-positive CU individuals and those with Aβ-positive MCI and AD dementia) with longitudinal [^18^F]flortaucipir tau PET (from the AVID 05e, Expedition-3, ADNI, and Sweden BioFINDER-1 cohorts) were included as a validation cohort. Inclusion and exclusion criteria are described elsewhere^42^; Aβ-status was determined using amyloid-PET (AVID 05e and Expedition-3, [^18^F]florbetapir; ADNI, [^18^F]florbetaben or [^18^F]florbetapir; BioFINDER, [^18^F]flutemetamol) and neocortical composite cutoffs (eMethods 4 in Supplement 1). Although the group-level ROIs were the same as those used with [^18^F]RO948, individualized ROIs were redefined using [^18^F]flortaucipir.

### Statistical Analyses

Annual percentage change in tau-PET SUVR was determined between baseline and follow-up within ROIs. This was calculated as the difference between follow-up and baseline, divided by baseline uptake and divided by the time interval between scans in years. Annual change in SUVR [(follow-up SUVR − baseline SUVR) / change in time] is reported in eTables 4 to 7 in Supplement 1. In order to test whether the annual changes in tau-PET SUVR were significant, 1-sample *t* tests against 0 were performed groupwise for each ROI. To determine the effect of ROI on sample size requirements in theoretical trials using tau PET as outcome, a power analysis was performed groupwise, assuming 20%, 30%, or 40% reductions in the annual percentage change in tau-PET SUVR compared with placebo. Differences between group-level and individualized ROIs—for both annual change in SUVR and sample size reductions—were tested using bootstrapping (n = 1000; ie, does the mean of the higher value exceed the 95% CI of the lower value). All analyses were done with R version, 4.2.1 (R Foundation).

## Results

### Participant Characteristics and Change in Tau PET

**Figure 3.**
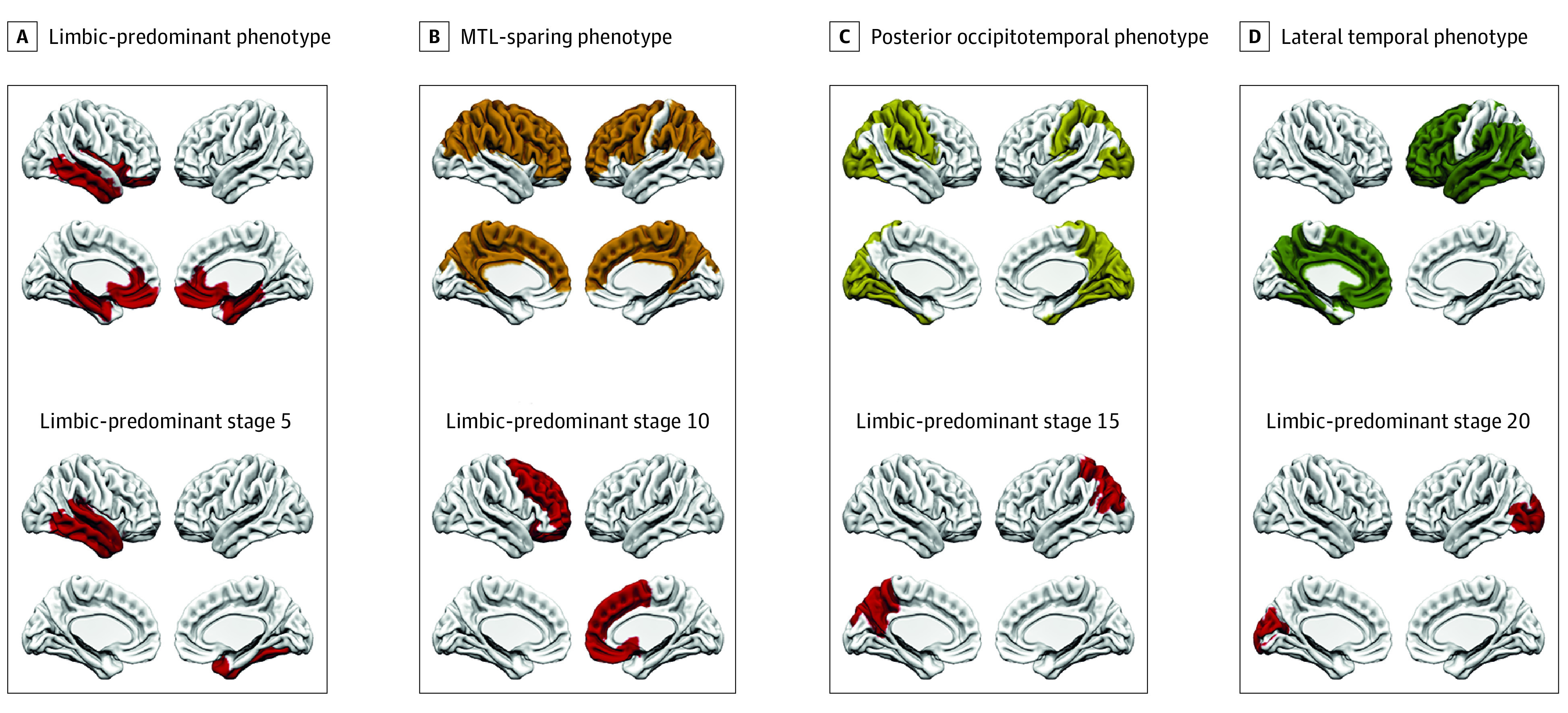
Subtype and Stage Inference Regions of Interest The 4-tau–positron emission tomography (PET) subtypes are shown. A, Subtype 1, limbic-predominant phenotype. B, Subtype 2, medial temporal lobe (MTL) sparing. C, Subtype 3, posterior occipitotemporal phenotype. D, Subtype 4, lateral temporal. A representation of different stages (5, 10, 15, 20) for subtype 1 is displayed.

A total of 215 participants (mean [SD] age, 71.4 (7.5) years; 111 male [51.6%]; 104 female [48.4%]) with longitudinal tau PET were included in this analysis: 97 Aβ-positive CU individuals (45%), 77 Aβ-positive individuals with MCI (36%), and 41 individuals with AD dementia (19%) from the BioFINDER-2 study. Mean (SD) follow-up time was 1.8 (0.3) years. Participant characteristics are summarized in the Table. Group-level ROIs are shown in Figure 1 whereas the approaches used to derive individualized ROIs are shown in Figure 2 and Figure 3.

**Table.** Characteristics of Participants in the Biomarkers For Identifying Neurodegenerative Disorders Early and Reliably 2 (BioFINDER-2) Cohort

Characteristic	Amyloid-β positive		AD dementia (n = 41)
	CU (n = 97)	MCI (n = 77)	
Age, mean (SD), y	68.89 (9.19)	72.39 (7.68)	72.57 (7.27)
Sex, No. (%)			
Male	51 (52.6)	42 (54.5)	18 (43.9)
Female	46 (47.4)	35 (45.5)	23 (56.1)
Education, mean (SD), y	12.38 (3.57)	13.11 (4.63)	11.70 (4.45)
MMSE score, mean (SD)	28.68 (1.45)	26.81 (1.97)	23.39 (1.82)
Tau PET, mean (SD) scan interval, y	1.83 (0.36)	1.92 (0.53)	1.66 (0.34)

Abbreviations: AD, Alzheimer disease; CU, cognitively impaired; MCI, mild cognitive impairment; MMSE, Mini-Mental State Examination; PET, positron emission tomography.

A total of 406 participants with longitudinal [^18^F]flortaucipir tau PET composed the validation sample: 137 Aβ-positive CU individuals, 144 with Aβ-positive MCI, and 125 with AD dementia. The validation sample included participants from the following study cohorts AVID 05e (n = 151), Expedition-3 (n = 82), ADNI (n = 117), and Sweden BioFINDER-1 (n = 56).

**Figure 4.**
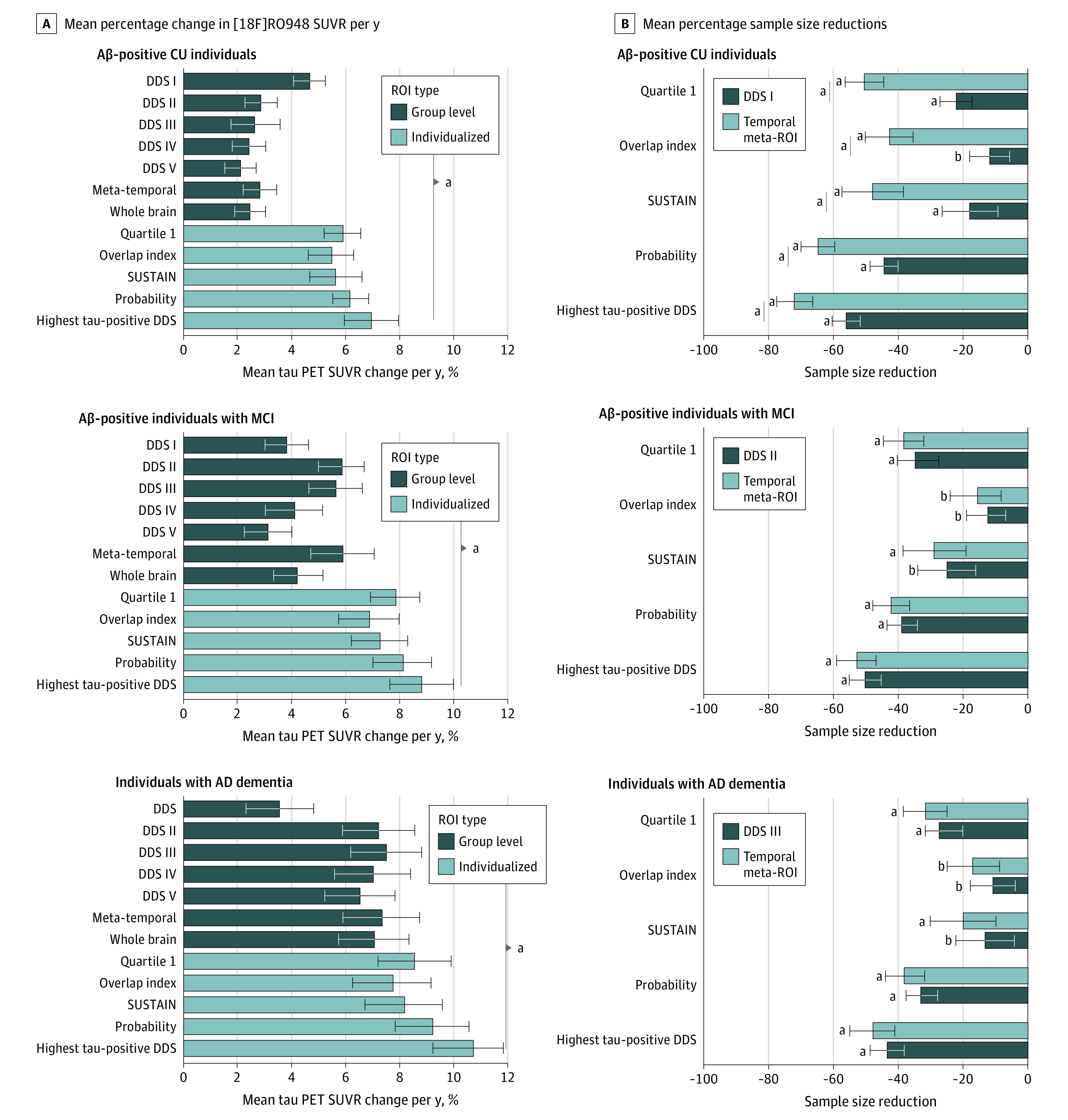
Annual Percentage Change in Tau–Positron Emission Tomography (PET) Standardized Uptake Value Ratio (SUVR) and Sample Size Reductions in a Theoretical Clinical Trial Using Tau-PET as an End Point Using Different Regions of Interest (ROIs) A, Mean percentage change in [^18^F]RO948 SURV per year along with 95% CIs for group level and individualized ROIs. *P* values are shown groupwise for comparisons between individualized ROIs and the best-performing group-level ROI. B, Mean percentage sample size reductions along with 95% CIs are shown for group-level and individualized ROIs compared with the temporal meta-ROI and best-performing data-driven ROIs (ie, data-driven stage I in amyloid β (Aβ)–positive cognitively unimpaired [CU]; data-driven stage II in Aβ-positive mild cognitive impairment [MCI]; data-driven stage III in Alzheimer disease [AD] dementia). *P* values are shown for each bar for comparisons against 0. Comparisons in sample reductions between temporal and the best-performing data-driven stage were only significant in Aβ-positive CU individuals (ie, sample size reductions were significantly greater in Aβ-positive CU individuals using data driven stage I, compared with the temporal meta-ROI). ^a^*P* <.001. ^b^*P* <.01.

Using group-level ROIs, the largest annual percentage increase in [^18^F]RO948 SUVR in Aβ-positive CU individuals was seen in DDS I (entorhinal cortex, hippocampus, and amygdala: 4.29%; 95% CI, 3.42%-5.16%; *P* < .001) (Figure 4A). In Aβ-positive individuals with MCI, the greatest change was seen in DDS II (temporal cortical regions: 5.82%; 95% CI, 4.67%-6.97%; *P* < .001) (Figure 4A), whereas in individuals with AD dementia, the greatest change was seen in stage III (parietal regions: 7.47%; 95% CI, 6.18%-8.78%; *P* < .001) (Figure 4A; eTables 4 and 5 in Supplement 1). Findings from 1-sample *t* tests are included in the eTables 4, 5, 6, and 7 in Supplement 1. Findings using Braak ROIs were similar to those using DDSs (eTables 6 and 7 in Supplement 1). Across both data-driven and Braak ROIs, variation in the number of tau-positive ROIs decreased across clinical groups (eTable 8 in Supplement 1). Estimates of annual change in SUVR were generally numerically higher using individualized ROIs (Figure 4A). However, significance levels varied by ROI and disease stage (eTable 9 in Supplement 1). Although all individualized ROIs outperformed group-level DDS I in Aβ-positive CU participants (DDS I, 4.29%; 95% CI, 3.42%-5.16%; overlap index, 5.14%; 95% CI, 4.29%-6.13%; SUSTAIN, 5.32%; 95% CI, 4.33%-6.31%; probability, 5.89%; 95% CI, 5.23%-6.84%; highest tau-PET–positive DDS, 6.69%; 95% CI, 5.66%-7.54%; all *P* <.001), only the probability and highest tau-PET–positive DDS approaches resulted in significantly higher estimates of annual percentage change in [^18^F]RO948 SUVR compared with the best-performing group-level data-driven ROI across Aβ-positive CU individuals and those with MCI (DDS II, 5.82%; 95% CI, 4.67%-6.97%; probability, 7.92%; 95% CI, 6.82%-9.02%; highest tau-PET–positive DDS, 8.67%; 95% CI, 7.49%-9.85%; *P* <.001) and AD dementia (DDS III, 7.47%; 95% CI, 5.98%- 8.96%; probability, 9.20%; 95% CI, 7.95%-10.45%; highest tau-PET–positive DDS, 10.74%; 95% CI, 9.33%-11.84%; *P* <.001).

Using individualized ROIs, the highest tau-PET–positive DDS approach performed best across diagnostic groups (Aβ-positive CU, 6.69%; 95% CI, 5.83%- 7.55%; *P* < .001; Aβ-positive MCI, 8.67%; 95% CI, 7.49%-9.85%; *P* < .001; AD dementia, 10.74%; 95% CI, 9.33%-12.20%; *P* < .001) (eTable 9 in Supplement 1), even when using an alternative reference region combining the cerebellum, brainstem, and eroded subcortical white matter (eFigure in Supplement 1).

### Power Analysis for Hypothetical Clinical Trials

Having compared annual percentage change estimates for [^18^F]RO948 using group-level and individualized ROIs, we estimated sample size requirements for a simulated intervention that reduced longitudinal tau accumulation by 30%. For each group (ie, Aβ-positive CU, Aβ-positive MCI, and AD dementia), sample size reductions for group-level and individualized ROIs were estimated compared with the temporal meta-ROI due to its widespread use with tau PET in the AD field and the best-performing group-level ROI (Figure 4B). In Aβ-positive CU individuals, sample size reductions for the individualized ROIs ranged from 42.74% (overlap index, 95% CI, 35.44%-49.74%; *P* < .001) to 72.10% (highest tau-PET positive DDS, 95% CI, 67.10%-77.20%; *P* < .001) compared with the temporal meta-ROI and between 12.21% (overlap index, 95% CI, 5.91%-18.51%; *P* = .01) and 56% (highest tau-PET–positive DDS, 95% CI, 51.17%-60.10%; *P* < .001) compared with DDS I (Figure 4B). A similar pattern was seen in Aβ-positive MCI sample size reductions between 15.94% (overlap index, 95% CI, 8.14%-23.74%; *P* = .001) and 53.02% (highest tau-PET–positive DDS, 95% CI, 47.10%-59.87%; *P* < .001) compared with temporal meta-ROI and between 12.22% (overlap index, 95% CI, 4.42%-18.10%; *P* = .01) and 50.38% (highest tau-PET–positive DDS, 95% CI, 45.43%-55.34%; *P* < .001) compared with DDS II (Figure 4B). In AD dementia, sample size reductions varied between 16.83% (overlap index, 95% CI, 8.78%-25.61%; *P* = .002) and 48.30% (highest tau-PET–positive DDS, 95% CI, 41.03%-89.10%; *P* < .001) compared with temporal meta-ROI and between 9.73% (overlap index, 95% CI, 2.68%-12.41%; *P* = .004) and 43.59% (highest tau-PET–positive DDS, 95% CI, 38.39%-81.98%; *P* < .001) compared with DDS III (Figure 4B). Only in Aβ-positive CU individuals were significant differences seen between sample size reductions using the temporal meta-ROI and best-performing DDS (quartile 1: temporal meta-ROI, 50.56%; 95% CI, 44.63%-56.50%; DDS I, 22%; 95% CI, 17.10%-26.94%; *P* <.001; overlap index: temporal meta-ROI, 42.74%; 95% CI, 35.44%-50.03%; DDS I, 11.65%; 95% CI, 5.36%-17.95%; *P* <.001; SUSTAIN; temporal meta-ROI, 47.88%; 95% CI, 38.45%-57.30%; DDS I, 17.76%; 95% CI, 9.34%-26.18%; *P* <.001; probability based: temporal meta-ROI, 64.78%; 95% CI, 59.67%-69.89%; DDS I, 44.43%; 95% CI, 40.32%-48.54%; *P* <.001; highest tau-PET–positive DDS: temporal meta-ROI, 72.10%; 95% CI, 66.60%-77.50%; DDS I, 55.90%; 95% CI, 51.45%-60.35%; *P* <.001) (Figure 4B). Sample size estimations (ie, the number of participants required per arm to detect an intervention effect) assuming hypothetical intervention effects of 20%, 30%, and 40% are included in eTables 10, 11, and 12 in the Supplement.

### Power and Sensitivity Analysis Comparing SUVR and Tau Extent

When performing a sensitivity analysis comparing annual percentage change in tau extent and [^18^F]RO948 SUVR in the temporal meta-ROI in a simulated intervention that reduced longitudinal tau accumulation by 30%, sample sizes were consistently lower for SUVR (Aβ-positive CU, 17.90%; 95% CI, 11.47%-24.33%; *P* = .007; Aβ-positive MCI, 29.47%; 95% CI, 19.55%-39.39%; *P* = .003; AD dementia, 33.94%; 95% CI, 25.02%-42.86%; *P* < .001) (eTable 13 in Supplement 1).

### Independent Validation Sample

Among the 406 participants (137 Aβ-positive CU, 144 Aβ-positive MCI, and 41 AD dementia) with longitudinal [^18^F]flortaucipir tau PET, the mean (SD) age was 77.18 (7.74) years, and 41 were female (48.32%) (eTable 14 in Supplement 1). Similar to findings in BioFINDER-2, the largest annual increase in [^18^F]flortaucipir SUVR was seen in stage I (3.88%; 95% CI, 3.17%-4.58%) in Aβ-positive CU individuals, with Aβ-positive individuals with MCI and AD dementia showing the largest increases in stages II (5.41%; 95% CI, 4.51%-6.31%) and III (6.08%; 95% CI, 4.90%-7.26%), respectively (eTables 15, 16, 17, and 18 in Supplement 1). Further, individualized ROIs resulted in significantly higher estimates of annual percentage change compared with group-level ROIs, with the highest tau-PET–positive DDS approach performed best across diagnostic groups (eTables 15 and 16 in Supplement 1). Similar to [^18^F]RO948, sample size reductions were greater using individualized ROIs compared with group-level ROIs, with highest tau-PET–positive DDS performing best (eTables 19, 20, and 21 in Supplement 1).

## Discussion

In this cohort study, we compared 2 approaches to defining ROIs for use with longitudinal tau PET: a group-level approach, where the same ROI was used for each participant, and an individualized approach where each participant received their own specific ROI. Our main finding suggests that individualized ROIs increased the sensitivity to detect longitudinal tau accumulation as well as intervention effects in simulated clinical trials using tau PET as outcome. The added value of the individualized ROIs—in terms of sensitivity to change in tau-PET signal over time and required sample size—was greater in the Aβ-positive CU group, compared with the Aβ-positive MCI and AD dementia groups. Importantly, among the methods used to generate the individualized ROIs, the simplest approach (highest tau-PET–positive DDS, where change in tau PET was calculated in the highest tau-PET–positive DDS) performed best, providing the highest tau-PET change estimates and sample size reductions.

Findings using group-level data-driven and PET-based Braak ROIs were consistent with previous longitudinal tau-PET studies in that tau accumulation was primarily seen in the medial temporal lobe early in the disease process (ie, DDS I and Braak I/II in Aβ-positive CU individuals) and primarily in temporal and parietal cortical regions (ie, DDSs II/III and Braak III/IV) in Aβ-positive individuals with cognitive impairment,^13,38,43,44,45,46^ consistent with the hierarchical Braak staging model of tau progression.^5,47,48^ The greater sensitivity of individualized assessment of tau accumulation over spatially predefined ROIs (eg, data-driven or Braak ROIs) across groups, however, suggests the view that tau deposition can be heterogeneous in AD.^7,8,9,10^ Variation in the number of tau-PET–positive data-driven or Braak ROIs showed that the regional heterogeneity of tau accumulation decreased with disease progression. This observation, combined with the fact that the differences in sample size reductions between individualized and groupwise ROIs were only significant in Aβ-positive CU individuals, suggests that the utility of individualized ROIs may be greatest at the preclinical stage of AD. Heterogeneity in tau deposition may be associated with varying tau starting sites (ie, epicenters) and the subsequent spread of tau via connections between brain regions. Previous work using [^18^F]flortaucipir found that tau accumulation rates were highest in brain regions with the closest connectivity-based proximity to tau epicenters (ie, Q1)^20^; here, we reproduced these findings and extended them through our comparison with DDSs. A similar explanation may also apply to the SUSTAIN-based subtypes in that the distinct tau-PET patterns seen across the 4 different subtypes resembled macroscale neuronal networks seeded from different regions within the temporal lobe.^16^ On the basis of earlier work showing that tau pathology may plateau or decrease with more advanced disease stage,^49^ contributing to interindividual variation in longitudinal change, the probability-based approach was an attempt to exclude both low-probability regions unlikely to show tau accumulation and high-probability regions thought to be at or approaching their maximum possible concentrations of tau.^49,50^

Consistent with the hierarchical aggregation characteristic of the Braak staging system for tau,^5,47,48^ we found that tau accumulation was greatest using the highest DDS showing abnormal [^18^F]RO948 SUVR levels (highest tau-PET–positive DDS). Though tau-PET positivity and pathological tau accumulation were also seen in the stages preceding the highest abnormal stage, suggesting that although there is an increasing burden of tau across the brain—as opposed to the spread of tau from one uninvolved area to the next—the accumulation of tau aggregates was greatest in the most recently affected brain region. In the context of clinical trials using tau PET as outcome, this approach would be comparatively straightforward to implement. Using the baseline tau-PET scan, prespecified tracer-specific cutoffs derived from a large academic cohort could be applied to either data-driven or Braak ROIs, with the highest tau-PET–positive ROI then selected for longitudinal use. However, further studies are required to assess the reproducibility of the thresholds for tau positivity presented here. In addition to its simplicity, the fact that the highest tau-PET–positive DDS approach was the best-performing ROI across groups suggests that it could be used in trials across the AD continuum. Although cognitive decline is typically considered as a primary outcome measure for clinical trials,^51^ recent work has highlighted the potential role of tau PET in clinical trials.^19,29^ This is further evidenced by antitau drugs entering the clinical trial pipeline.^52^ Although further comparative studies are required, due to large within- and between-patient variability in frequently used cognitive measures,^53^ tau PET may allow for AD trials of shorter duration and with fewer participants. Lastly, comparison of sample size reductions between annual percentage change in tau extent and SUVR in the temporal meta-ROI suggests that SUVR may be the preferred metric for longitudinal tau PET.

### Strengths and Limitations

Strengths of this study include that we compared several group-level and individualized approaches to defining ROIs, with the methods used for individualized ROIs varying in their level of complexity. Further, our study covered the clinical continuum of AD and had a comparatively long follow-up interval that was similar across groups. Importantly, we replicated our main findings with [^18^F]RO948 using longitudinal [^18^F]flortaucipir PET in an independent cohort. Limitations include the modest number of participants scanned with [^18^F]RO948 in the AD dementia group and the fact that we could not estimate within-participant measurement error because participants only had 2 tau-PET scans. This study contributes to the continuing investigation of optimized trial outcomes and precision medicine.^54^

## Conclusions

Findings of this cohort study suggest that individualized ROIs carried an advantage over group-level ROIs for assessing longitudinal tau changes and may increase the sensitivity to detect treatment effects in AD clinical trials using longitudinal tau-PET as an outcome. Future studies should assess additional methods for defining individualized ROIs.

## Notes

Supplement 1. **eMethods 1.** Inclusion and Exclusion Criteria for the Swedish BioFINDER-2 Study **eMethods 2.** Accounting for Off-Target Meningeal Binding of [^18^F]RO948 **eMethods 3.** Details for Group-Level and Individualized ROIs **eMethods 4.** Use of Amyloid-PET to Determine Aβ-Status in the [^18^F]Flortaucipir Cohort **eTable 1.** Annual Percent Change in [^18^F]RO948 SUVR Across Different Probability Intervals **eTable 2.** Regions Included in SUSTAIN-Based Individualized ROIs **eTable 3.** Annual Percent Change in [^18^F]RO948 SUVR Using Overlap Index and Masks Created Using Different Voxel-Wise Cutoffs **eTable 4.** Mean Annual Percent Change in [^18^F]RO948 Tau PET SUVR **eTable 5.** Mean Annual Change in [^18^F]RO948 Tau PET SUVR **eTable 6.** Mean Annual Percent Change in [^18^F]RO948 Tau PET SUVR Using Braak ROIs **eTable 7.** Mean Annual Change in [^18^F]RO948 Tau PET SUVR Using Braak ROIs **eTable 8.** Distribution of Tau PET Positivity Across Data-Driven and Braak ROIs Using [^18^F]RO948 **eTable 9.** Comparison of Mean Annual Change in [^18^F]RO948 Tau PET SUVR Between Group-Level and Individualized ROIs **eTable 10.** [^18^F]RO948-Based Sample Size Estimations for Group-Level and Individualized ROIs Assuming a 20% Intervention Effect **eTable 11.** [^18^F]RO948-Based Sample Size Estimations for Group-Level and Individualized ROIs Assuming a 30% Intervention Effect **eTable 12.** [^18^F]RO948-Based Sample Size Estimations for Group-Level and Individualized ROIs Assuming a 40% Intervention Effect **eTable 13.** Sensitivity Analysis Comparing Sample Size Reductions Between Tau Extent and Annual Percent Change in SUVR in the Temporal Meta-ROI Using [^18^F]RO948 **eTable 14.** Participant Characteristics in the [^18^F]Flortaucipir Validation Sample **eTable 15.** Mean Annual Percent Change in [^18^F]Flortaucipir Tau PET SUVR **eTable 16.** Mean Annual Change in [^18^F]Flortaucipir Tau PET SUVR **eTable 17.** Mean Annual Percent Change in [^18^F]Flortaucipir Tau PET SUVR Using Braak ROIs **eTable 18.** Mean Annual Change in [^18^F]Flortaucipir Tau PET SUVR Using Braak ROIs **eTable 19.** [^18^F]Flortaucipir-Based Sample Size Estimations for Group-Level and Individualized ROIs Assuming a 20% Intervention Effect **eTable 20.** [^18^F]Flortaucipir-Based Sample Size Estimations for Group-Level and Individualized ROIs Assuming a 30% Intervention Effect **eTable 21.** [^18^F]Flortaucipir-Based Sample Size Estimations for Group-Level and Individualized ROIs Assuming a 40% Intervention Effect **eFigure.** Sensitivity Analysis for [^18^F]RO948 Using the Cerebellum, Brain Stem, and Eroded Subcortical White Matter as a Composite Reference Region

Supplement 2. Nonauthor Collaborators

Supplement 3. Data Sharing Statement
