# Supplementary material for: Comparison of Group-Level and Individualized Brain Regions for Measuring Change in Longitudinal Tau Positron Emission Tomography in Alzheimer Disease
Source: JAMA Neurol. 2023 May 8;80(6):614–23. doi: 10.1001/jamaneurol.2023.1067 (PMC10167602; doi:10.1001/jamaneurol.2023.1067)
Supplement: Supplement 1. — eMethods 1. Inclusion and Exclusion Criteria for the Swedish BioFINDER-2 Study eMethods 2. Accounting for Off-Target Meningeal Binding of [18F]RO948 eMethods 3. Details for Group-Level and Individualized ROIs eMethods 4. Use of Amyloid-PET to Determine Aβ-Status in the [18F]Flortaucipir Cohort eTable 1. Annual Percent Change in [18F]RO948 SUVR Across Different Probability Intervals eTable 2. Regions Included in SUSTAIN-Based Individualized ROIs eTable 3. Annual Percent Change in [18F]RO948 SUVR Using Overlap Index and Masks Created Using Different Voxel-Wise Cutoffs eTable 4. Mean Annual Percent Change in [18F]RO948 Tau PET SUVR eTable 5. Mean Annual Change in [18F]RO948 Tau PET SUVR eTable 6. Mean Annual Percent Change in [18F]RO948 Tau PET SUVR Using Braak ROIs eTable 7. Mean Annual Change in [18F]RO948 Tau PET SUVR Using Braak ROIs eTable 8. Distribution of Tau PET Positivity Across Data-Driven and Braak ROIs Using [18F]RO948 eTable 9. Comparison of Mean Annual Change in [18F]RO948 Tau PET SUVR Between Group-Level and Individualized ROIs eTable 10. [18F]RO948-Based Sample Size Estimations for Group-Level and Individualized ROIs Assuming a 20% Intervention Effect eTable 11. [18F]RO948-Based Sample Size Estimations for Group-Level and Individualized ROIs Assuming a 30% Intervention Effect eTable 12. [18F]RO948-Based Sample Size Estimations for Group-Level and Individualized ROIs Assuming a 40% Intervention Effect eTable 13. Sensitivity Analysis Comparing Sample Size Reductions Between Tau Extent and Annual Percent Change in SUVR in the Temporal Meta-ROI Using [18F]RO948 eTable 14. Participant Characteristics in the [18F]Flortaucipir Validation Sample eTable 15. Mean Annual Percent Change in [18F]Flortaucipir Tau PET SUVR eTable 16. Mean Annual Change in [18F]Flortaucipir Tau PET SUVR eTable 17. Mean Annual Percent Change in [18F]Flortaucipir Tau PET SUVR Using Braak ROIs eTable 18. Mean Annual Change in [18F]Flortaucipir Tau PET SUVR Using Braak ROIs eTable 19. [18 [file jamaneurol-e231067-s001.pdf]

Supplementary Online Content

Leuzy A, Binette AP, Vogel JW, et al; Alzheimer's Disease Neuroimaging Initiative. Comparison of group-level and individualized brain regions for measuring change in longitudinal tau positron emission tomography in Alzheimer disease. *JAMA Neurol*. Published online May 8, 2023. doi:10.1001/jamaneurol.2023.1067

eMethods 1. Inclusion and Exclusion Criteria for the Swedish BioFINDER-2 Study
eMethods 2. Accounting for Off-Target Meningeal Binding of [^{18}F]RO948
eMethods 3. Details for Group-Level and Individualized ROIs
eMethods 4. Use of Amyloid-PET to Determine Aβ-Status in the [^{18}F]Flortaucipir Cohort
eTable 1. Annual Percent Change in [^{18}F]RO948 SUVR Across Different Probability Intervals **eTable 2.** Regions Included in SUSTAIN-Based Individualized ROIs
eTable 3. Annual Percent Change in [^{18}F]RO948 SUVR Using Overlap Index and Masks Created Using Different Voxel-Wise Cutoffs
eTable 4. Mean Annual Percent Change in [^{18}F]RO948 Tau PET SUVR
eTable 5. Mean Annual Change in [^{18}F]RO948 Tau PET SUVR
eTable 6. Mean Annual Percent Change in [^{18}F]RO948 Tau PET SUVR Using Braak ROIs
eTable 7. Mean Annual Change in [^{18}F]RO948 Tau PET SUVR Using Braak ROIs
eTable 8. Distribution of Tau PET Positivity Across Data-Driven and Braak ROIs Using [^{18}F]RO948
eTable 9. Comparison of Mean Annual Change in [^{18}F]RO948 Tau PET SUVR Between Group-Level and Individualized ROIs
eTable 10. [^{18}F]RO948-Based Sample Size Estimations for Group-Level and Individualized ROIs Assuming a 20% Intervention Effect
eTable 11. [^{18}F]RO948-Based Sample Size Estimations for Group-Level and Individualized ROIs Assuming a 30% Intervention Effect
eTable 12. [^{18}F]RO948-Based Sample Size Estimations for Group-Level and Individualized ROIs Assuming a 40% Intervention Effect
eTable 13. Sensitivity Analysis Comparing Sample Size Reductions Between Tau Extent and Annual Percent Change in SUVR in the Temporal Meta-ROI Using [^{18}F]RO948
eTable 14. Participant Characteristics in the [^{18}F]Flortaucipir Validation Sample
eTable 15. Mean Annual Percent Change in [^{18}F]Flortaucipir Tau PET SUVR
eTable 16. Mean Annual Change in [^{18}F]Flortaucipir Tau PET SUVR
eTable 17. Mean Annual Percent Change in [^{18}F]Flortaucipir Tau PET SUVR Using Braak ROIs
eTable 18. Mean Annual Change in [^{18}F]Flortaucipir Tau PET SUVR Using Braak ROIs
eTable 19. [^{18}F]Flortaucipir-Based Sample Size Estimations for Group-Level and Individualized ROIs Assuming a 20% Intervention Effect
eTable 20. [^{18}F]Flortaucipir-Based Sample Size Estimations for Group-Level and Individualized ROIs Assuming a 30% Intervention Effect
eTable 21. [^{18}F]Flortaucipir-Based Sample Size Estimations for Group-Level and Individualized ROIs Assuming a 40% Intervention Effect
eFigure. Sensitivity Analysis for [^{18}F]RO948 Using the Cerebellum, Brain Stem, and Eroded Subcortical White Matter as a Composite Reference Region

© 2023 Leuzy A et al. *JAMA Neurology.*

eMethods 1. Inclusion and exclusion criteria for the Swedish BioFINDER-2 study

The BioFINDER-2 study enrolls participants in five sub-cohorts; Cohort A and B includes neurologically and cognitively healthy controls. The inclusion criteria are: i) ages 40-65 years (cohort A) and ages 66-100 years (cohort B); ii) absence of cognitive symptoms as assessed by a physician with special interest in cognitive disorders; iii) MMSE score 27-30 points (cohort A) or 26-30 points (cohort B) at screening visit; iv) do not fulfill the criteria for MCI or any dementia according to DSM-5[1]; v) fluent in Swedish. The recruitment process of cohorts A and B is designed to build two study populations with 50% *APOE* ε4 carriers in each.

Cohort C comprises participants with subjective cognitive decline (SCD) or minor neurocognitive impairment (MCI) (the latter according to DSM-5 (American Psychiatric Association,2013). Inclusion criteria are: i) Age 40-100 years; ii) referred to the memory clinics due to cognitive symptoms; iii) MMSE score of 24 – 30 points; iv) does not fulfill the criteria for any dementia (major neurocognitive disorder) according to DSM-5[1], v) fluent in Swedish. In accordance with the research framework by the National Institute on Aging-Alzheimer's Association[2] study participants with SCD were analyzed together with the cognitively healthy participants (and combined in the cognitively unimpaired group). Participants were classified as having MCI if they performed worse than -1.5 SD in any cognitive domain according to age and education stratified test norms. The neuropsychological battery covered the domains attention/executive function (Trail Making Test A and B, Symbol Digit Modalities Test, and AQT), memory (10 word immediate and delayed recall from the Alzheimer's Disease Assessment Scale [ADAS]), verbal ability (verbal fluency and the short version of the Boston Naming Test) and visuospatial function (incomplete letters and cube analysis from the Visual Object and Space Perception battery). Those that were not classified as MCI were considered to have SCD.

© 2023 Leuzy A et al. *JAMA Neurology.*

Cohort D consists of participants with dementia due to AD. Inclusion criteria are: i) Age 40-100 years; ii) referred to the memory clinics due to cognitive symptoms; iii) MMSE score of ≥12 points; iv) fulfill the DSM-5 criteria for dementia (major neurocognitive disorder) due to Alzheimer's disease[1]; v) fluent in Swedish. Exclusion criteria for all sub-cohorts are: i) significant unstable systemic illness that makes it difficult to participate in the study; ii) current significant alcohol or substance misuse; iii) refusing lumbar puncture, MRI or PET.

References

1. APA. Diagnostic Criteria from DSM-5. Washington, DC2013.
2. Jack CR, Jr., Bennett DA, Blennow K, et al. NIA-AA Research Framework: Toward a biological definition of Alzheimer's disease. *Alzheimers Dement*. 2018;14(4):535-562.

© 2023 Leuzy A et al. *JAMA Neurology.*

eMethods 2. Accounting for off-target meningeal binding of [^{18}F]RO948

[^{18}F]RO948 tau PET scans were performed on a digital GE Discovery MI scanner (General Electric Medical Systems), with LIST mode emission data acquired 70–90 min post injection. Low-dose CT scans were performed immediately prior to the PET scans for attenuation correction. PET data was reconstructed using VPFX-S (ordered subset expectation maximization (OSEM) with time-of-flight (TOF) and point spread function (PSF) corrections) with 6 iterations and 17 subsets. In order to account for meningeal binding seen with [^{18}F]RO948, no smoothing was performed during the reconstruction process. LIST mode data was then binned into 4 x 5-min time frames, and the resulting PET images motion corrected, summed, and co-registered to their corresponding T1-weighted MR images. To further improve GM delineation and by extension reduce bleed-in from the meningeal signal, a refined meningeal segmentation was calculated combining T1 and T2-FLAIR images (using the Samseg tool, part of the FreeSurfer v7.0 program suite). The resulting binary mask was then used to null voxels in the atlas produced in the FreeSurfer segmentation, thus pruning away signal from meningeal voxels erroneously identified as being part of the given GM region.

© 2023 Leuzy A et al. *JAMA Neurology.*

eMethods 3. Details for group-level and individualized ROIs

Data driven stages

In order to generate the regions of interest used in the primary analyses, a two-component Gaussian mixture model was first applied to all available baseline [^{18}F]RO948 SUVR data (cognitively unimpaired, n=464; mild cognitive impairment, n=196; AD dementia, n=150; non-AD disorders, n=216).[1] For each FreeSurfer ROI, the SUVR value was converted to a tau-positive probability (i.e., the probability that a subjects' SUVR within that ROI value fell within the rightmost portion of the Gaussian distribution, representing abnormal signal). Recursive k-means clustering (n=1000) was then performed on the bootstrapped samples of the input probability data. After each clustering iteration, information about cluster membership is stored in the form of an adjacency matrix. The adjacency matrices were averaged resulting in a stability matrix representing probabilities of different regions clustering together. Lastly, hierarchical agglomerative clustering with Ward criterion was applied to the stability matrix in order to obtain the final clustering solution, with five-cluster solution adopted based on silhouette scores. Identified clusters were then submitted to an event-based model (EBM)[2] to determine in which order the identified clusters become abnormal. In EBM, an 'event' represents the transition from a normal to an abnormal state, with the EBM determining the event sequence that maximizes the data likelihood (i.e., the most likely ordering of the events). Regional tau-positive probabilities were averaged within cluster-derived ROIs and submitted to EBM, using 10,000 Monte-Carlo simulations to derivate uncertainty in event ordering.

Data-driven stages were as follows[1]: Stage I, amygdala, hippocampus, entorhinal cortex; Stage II, banks of superior temporal sulcus, fusiform gyrus, inferior temporal cortex, middle temporal gyrus, parahippocampus, superior temporal cortex, temporal pole; Stage III, caudal middle frontal cortex, inferior parietal cortex, isthmus cingulate, lateral occipital cortex, posterior cingulate cortex, precuneus, superior parietal cortex, supramarginal cortex; Stage IV,

caudal anterior cingulate, frontal pole, insula, lateral orbitofrontal cortex, medial orbitofrontal cortex, parsopercularis, parsorbitalis, pars triangularis, rostral anterior cingulate, rostral middle frontal cortex, superior frontal cortex; Stage V, cuneus, lingual gyrus, paracentral cortex, pericalcarine cortex, postcentral cortex, precentral postcentral cortex, transverse temporal cortex.

References

1. Leuzy A, Smith R, Cullen NC, et al. Biomarker-Based Prediction of Longitudinal Tau Positron Emission Tomography in Alzheimer Disease. *JAMA Neurol*. 2021.
2. Fonteijn HM, Modat M, Clarkson MJ, et al. An event-based model for disease progression and its application in familial Alzheimer's disease and Huntington's disease. *Neuroimage*. 2012;60(3):1880-1889.

© 2023 Leuzy A et al. *JAMA Neurology.*

Temporal and whole brain meta-ROIs

The meta-temporal ROI includes the entorhinal cortex, amygdala, parahippocampus, fusiform gyrus, inferior temporal cortex and middle temporal cortex. The whole brain meta-ROI included all 41 allocortical and isocortical FreeSurfer ROIs.[1]

References

1. Jack CR, Jr., Wiste HJ, Weigand SD, et al. Defining imaging biomarker cut points for brain aging and Alzheimer's disease. Alzheimers Dement. 2017;13(3):205-216.

© 2023 Leuzy A et al. *JAMA Neurology.*

Braak stages

Tau-PET based Braak stages were derived using a Z-score based approach, as described by Cho and colleagues.[1] Regional mean and SD values for 25 FreeSurfer-based cortical regions were determined using 45 amyloid-negative (neocortical [^{18}F]florbetaben SUVR < 1.4) controls whose [^{18}F]flortaucipir SUVR values in the entorhinal cortex were < 1.2. After calculating Z scores for all participants, the authors sorted the 25 regions in descending order by the number of participants whose regional Z score values exceeded 2.5 for each region. Participants were also sorted by the extent of regional involvement. After sorting all participants in ascending order by the number of regions that had Z score values>2.5, participants were then sorted by the mean Z score values across all regions. Differences in the frequency of regional involvement across the regions was assessed using bootstrapping (n=1000), with asymptotic p-values (Bonferroni corrected) calculated for all combination of regional pairs. Reproducibility of the tau spreading order was tested using 10 SUVR cut-offs (2.1–3.0).

Having determined the spreading order of tau pathology, the 25 FreeSurfer-based cortical regions were classified into Braak tau stages, maintaining the identified spreading order. For each Braak stage, volume-weighted average Z scores of the composite regions were used. Braak ROIs were as follows: Braak I, entorhinal cortex; Braak II, hippocampus; Braak III, amygdala, fusiform gyrus, parahippocampus; Braak IV, inferior temporal, middle temporal cortex; Braak V, caudal anterior cingulate, caudal middle frontal, frontal pole, inferior parietal, insula, lateral occipital, lateral orbitofrontal, lingual, medial orbitofrontal, posterior cingulate, precuneus, rostral anterior cingulate, rostral middle frontal, superior frontal, superior parietal, superior temporal, supramarginal gyrus; Braak VI, cuneus, paracentral, pericalrine, postcentral and precentral gyrus. As the hippocampus has been shown to be affected by off-target binding using [^{18}F]flortaucipir,[2] the hippocampus was not included in the staging .

© 2023 Leuzy A et al. *JAMA Neurology.*

References

1. Cho H, Choi JY, Hwang MS, et al. In vivo cortical spreading pattern of tau and amyloid in the Alzheimer disease spectrum. *Ann Neurol*. 2016;80(2):247-258.

2. Marquie M, Normandin MD, Vanderburg CR, et al. Validating novel tau positron emission tomography tracer [F-18]-AV-1451 (T807) on postmortem brain tissue. *Ann Neurol*. 2015;78(5):787-800.

© 2023 Leuzy A et al. *JAMA Neurology.*

Probability-based approach

On the basis of earlier work showing that tau pathology may plateau or decrease with more advanced disease stage,[1] contributing to interindividual variation in longitudinal change, the probability-based approach was an attempt to exclude both low probability regions unlikely to show tau accumulation and high probability regions though to be at or approaching their maximum possible concentrations of tau.[2] Annual change in [^{18}F]RO948 SUVR across different probability intervals is summarized in eTable 1.

References

1. Phillips JS, Nitchie FJt, Da Re F, et al. Rates of longitudinal change in (18) F-flortaucipir PET vary by brain region, cognitive impairment, and age in atypical Alzheimer's disease. Alzheimers Dement. 2022;18(6):1235-1247.

2 Whittington A, Gunn RN, Alzheimer's Disease Neuroimaging I. Tau(IQ): A Canonical Image Based Algorithm to Quantify Tau PET Scans. J Nucl Med. 2021;62(9):1292-1300.

© 2023 Leuzy A et al. *JAMA Neurology.*

Overlap Index

Overlap Index: a recently proposed method that assesses the stability of voxels above a defined threshold, based on the assumption that suprathreshold voxels that remain stable over time represent true positive signal due to tau pathology and not random variability.[1] In order to calculate the Overlap Index, baseline and follow-up scans must first be binarized using cut-offs to create masks that can then be used to determine spatial overlap. In the original publication, an SUVR cut-off of > 1.40 was used. We tested a range of cut-offs (1.30, 1.35, 1.40 and 1.45) though also found that 1.40 worked best in that it resulted in the highest annual percent change in tau PET SUVR. Once the masks had been established using this cut-off, a cluster filter was applied to remove all clusters with less than 20 voxels. Annual change in [^{18}F]RO948 SUVR using masks created using the different cut-offs are summarized in eTable 3.

References

1. Lee J, Burkett BJ, Min HK, et al. The Overlap Index as a means of evaluating early tau-PET signal reliability. J Nucl Med. 2022.

© 2023 Leuzy A et al. *JAMA Neurology.*

Subtype and Stage Inference

Subtype and Stage Inference (SUSTAIN) is an unsupervised machine-learning technique that can identify subgroups with common patterns of disease progression.[1] Combining clustering with disease progression modeling, SUSTAIN first determines the number of subtypes within the dataset followed by the stages that best define each subtype. Each participant is then assigned a probability for belonging to each subtype and stage.

SUSTAIN models linear transition across discrete points along a progression of indices of severity (*z*-scores) separately across different ROIs. Input therefore requires a subject *x* feature matrix combining the mean tau PET signal and associated severity scores within different ROIs. In order to define cut-offs for severity scores, SUVR values were first normalized to account for differences in PET signal between regions. Two-component Gaussian mixture models were then applied to define normal and abnormal distributions within each ROI. Tau z-scores were then created by normalizing values using mean of the normal distribution. For all ROIs, Z score values of 2, 5 and 10 were arbitrarily selected as severity score control points.

In the present work, SUSTAIN was applied using a four-subtype solution previously found to provide the best model fit using a large sample of [^{18}F]flortaucipir tau-PET data.[2] The four subtype model was applied to probabilistically assign individuals to 1 of 30 progressive stages along 1 of the 4 subtype trajectories. In order to create an individualized ROI for each participant, the FreeSurfer regions included in each stage were grouped as follows: 1-10, 11-20 and 21-30. The ROIs belonging to each stage are included in eTable 2.

References

1. Young AL, Marinescu RV, Oxtoby NP, et al. Uncovering the heterogeneity and temporal complexity of neurodegenerative diseases with Subtype and Stage Inference. *Nat Commun*. 2018;9(1):4273.

© 2023 Leuzy A et al. *JAMA Neurology.*

2. Vogel JW, Young AL, Oxtoby NP, et al. Four distinct trajectories of tau deposition identified in Alzheimer's disease. *Nat Med*. 2021;27(5):871-881.

© 2023 Leuzy A et al. *JAMA Neurology.*

Power Analysis – Sensitivity Analysis using Tau Extent

Tau Extent[1]—defined as the number of voxels above a defined threshold, divided by the total number of voxels in that region (i.e., the percentage of abnormal voxels within a given region)—was calculated for the temporal meta-ROI using an SUVR cut-off > 1.36, derived using a voxelwise implementation of the Youden's index (AD dementia [n=144] vs Aβ-negative CU [n=259].

References

1. Sanabria Bohorquez S, Marik J, Ogasawara A, et al. [(18)F]GTP1 (Genentech Tau Probe 1), a radioligand for detecting neurofibrillary tangle tau pathology in Alzheimer's disease. *Eur J Nucl Med Mol Imaging*. 2019;46(10):2077-2089.

© 2023 Leuzy A et al. *JAMA Neurology.*

eMethods 4. Use of amyloid-PET to determine Aβ-status in the [^{18}F]flortaucipir cohort

In the ADNI, Avid 05e and Expedition-3 cohort, Aβ-status was determined using [^{18}F]florbetapir and a cortical composite SUVR cut-offs of >1.11 (ADNI) or >1.10 (Avid 05e and Expedition-3) using the whole cerebellum as reference region.[1] In BioFINDER, Aβ-status was determined using [^{18}F]flutemetamol and a cortical composite cut-off of >0.743 with a white matter/brainstem/cerebellar reference region.[2,3]

References

1. Pontecorvo MJ, Arora AK, Devine M, et al. Quantitation of PET signal as an adjunct to visual interpretation of florbetapir imaging. *Eur J Nucl Med Mol Imaging*. 2017;44(5):825-837.
2. Thurfjell L, Lilja J, Lundqvist R, et al. Automated quantification of 18F-flutemetamol PET activity for categorizing scans as negative or positive for brain amyloid: concordance with visual image reads. *J Nucl Med.* 2014;55(10):1623-1628.
3. Palmqvist S, Scholl M, Strandberg O, et al. Earliest accumulation of beta-amyloid occurs within the default-mode network and concurrently affects brain connectivity. *Nat Commun*. 2017;8(1):1214.

eTable 1. Annual percent change in [^{18}F]RO948 SUVR across different probability intervals

	Mean annual change [95% CI]		
**Probability interval**	**Aβ-positive CU**	**Aβ-positive MCI**	**AD dementia**
0.2-1	3.74 [2.53, 4.95]	5.93 [4.78, 7.08]	7.08 [5.70, 8.48]
0.2-9	3.76 [2.57, 4.95]	6.07 [4.92, 7.21]	6.57 [5.02, 8.12]
0.2-0.8	3.80 [2.63, 4.97]	5.97 [4.85, 7.09]	6.69 [5.14, 8.24]
0.2-0.7	3.83 [2.83, 4.83]	6.13 [5.01, 7.25]	6.58 [4.99, 8.17]
0.3-1	4.08 [2.83, 5.33]	6.25 [4.96, 7.54]	6.46 [4.88, 8.06]
0.3-9	4.10 [2.88, 5.32]	6.47 [5.24, 7.70]	6.89 [5.26, 8.52]
0.3-0.8	4.11 [2.88, 5.32]	6.61 [5.39, 7.83]	7.24 [5.57, 8.91]
0.3-0.7	4.14 [2.92, 5.36]	6.58 [5.36, 7.80]	7.22 [5.51, 8.93]
0.4-1	5.39 [4.56, 6.22]	6.37 [5.04, 7.70]	7.45 [4.85, 8.05]
0.4-9	5.66 [4.44, 6.88]	6.89 [5.63, 8.15]	7.93 [5.23, 8.63]
0.4-0.8	5.89 [4.69, 7.10]	7.89 [6.69, 9.09]	9.20 [7.58, 10.8]
0.4-0.7	5.73 [4.57, 6.89]	7.81 [6.61, 9.03]	8.34 [5.72, 10.9]

© 2023 Leuzy A et al. *JAMA Neurology.*

Aβ indicates amyloid-β; CU, cognitively unimpaired; MCI, mild cognitive impairment; AD, Alzheimer disease

© 2023 Leuzy A et al. *JAMA Neurology.*

eTable 2. Regions included in SUSTAIN-based individualized ROIs

	**Subtype 1**	**Subtype 2**	**Subtype 3**	**Subtype 4**
**Stages 1-10**	R_MTL_5<br>L_MTL_5<br>R_Temporal_2<br>L_Temporal_2<br>L_Parietal_2<br>R_Parietal_2<br>L_Frontal_2<br>R_Frontal_2	R_MTL_2<br>L_MTL_2<br>R_Temporal_2<br>L_Temporal_2<br>R_Frontal_2<br>L_Frontal_2<br>L_Parietal_5<br>R_Parietal_5	R_MTL_2<br>L_MTL_2<br>R_Temporal_2<br>R_Occipital_2<br>L_Parietal_2<br>L_Occipital_5<br>L_Temporal_5<br>R_Parietal_2	L_MTL_2<br>R_MTL_2<br>L_Temporal_5<br>L_Parietal_2<br>L_Occipital_2<br>L_Frontal_2<br>R_Parietal_2<br>R_Temporal_5
**Stages 11-20**	L_Temporal_5<br>R_Parietal_5<br>L_Parietal_5<br>R_Temporal_10<br>R_Occipital_5<br>L_Occipital_5	L_Temporal_5<br>R_Temporal_5<br>L_Parietal_10<br>R_Parietal_10<br>R_Occipital_5<br>L_Occipital_5<br>L_Frontal_5<br>R_Frontal_5	R_Temporal_5<br>R_Occipital_10<br>L_Occipital_10<br>L_Parietal_10<br>R_Parietal_10<br>R_Frontal_2<br>L_Frontal_2	L_Temporal_10<br>L_MTL_5<br>L_Parietal_10<br>L_Frontal_5<br>R_Frontal_5<br>L_Frontal_10<br>R_Parietal_10
**Stages 21-30**	L_Temporal_10<br>R_Parietal_10<br>L_Parietal_10<br>R_Occipital_10<br>R_Frontal_10<br>L_Frontal_10<br>R_MTL_10<br>L_Occipital_10<br>L_MTL_10	R_Temporal_10<br>L_Temporal_10<br>R_Frontal_10<br>R_Occipital_10<br>L_Frontal_10<br>L_Occipital_10<br>L_MTL_10<br>R_MTL_10	L_Temporal_10<br>R_Temporal_10<br>L_MTL_10<br>R_MTL_10<br>R_Frontal_10<br>L_Frontal_10	R_Temporal_10<br>R_Frontal_10<br>R_MTL_5<br>R_MTL_10<br>L_Occipital_10<br>R_Occipital_10<br>L_MTL_10

© 2023 Leuzy A et al. *JAMA Neurology.*

Subtype 1: limbic-predominant phenotype; Subtype 2: medial temporal lobe sparing; Subtype 3: posterior occipitotemporal phenotype; Subtype 4: lateral temporal. R=Right hemisphere; L=Left hemisphere; 2, 5 and 10 refer to Z score severity scores. For each composite ROI, individual ROIs were weighted by their severity scores and divided by the total number of included regions. MTL=medial temporal lobe: entorhinal cortex, hippocampus, amygdala, parahippocampal gyrus; Temporal: inferior temporal cortex, middle temporal cortex, superior temporal cortex, fusiform gyrus, temporal pole; Parietal: inferior parietal cortex, superior parietal cortex, precuneus; Frontal: caudal middle frontal cortex, lateral orbitofrontal cortex, medial orbital frontal cortex, rostral middle frontal cortex, superior frontal cortex, frontal pole; Occipital: lateral occipital cortex, cuneus.

© 2023 Leuzy A et al. *JAMA Neurology.*

eTable 3. Annual percent change in [^{18}F]RO948 SUVR using Overlap Index and masks created using different voxel-wise cut-offs

	**Mean annual change [95% CI]**		
**SUVR cut-off**	**Aβ-positive CU**	**Aβ-positive MCI**	**AD dementia**
> 1.30	3.81 [3.05, 4.57]	5.71 [4.76, 6.67]	6.39 [4.77, 8.10]
> 1.35	3.92 [3.14, 4.70]	5.52 [4.52, 6.49]	6.85 [5.20, 8.50]
> 1.40	5.14 [4.18, 6.10]	6.61 [5.28, 7.94]	7.74 [6.15, 9.33]
> 1.45	4.84 [3.72, 5.96]	5.96 [4.91, 7.01]	6.97 [5.29, 8.65]

Aβ indicates amyloid-β; CU, cognitively unimpaired; MCI, mild cognitive impairment; AD, Alzheimer disease; SUVR, Standardized uptake value ratio.

© 2023 Leuzy A et al. *JAMA Neurology.*

eTable 4. Mean annual percent change in [^{18}F]RO948 tau PET SUVR

	**Mean annual change [95% CI]**		
**ROI**	**Aβ-positive CU**	**Aβ-positive MCI**	**AD dementia**
Data-driven stage I	4.29 [3.42, 5.16] ***	3.67 [2.55, 4.79] ***	3.41 [2.22, 4.85] ***
Data-driven stage II	2.42 [1.80, 3.04] *	5.82 [4.67, 6.97] ***	7.20 [5.85, 8.55] ***
Data-driven stage III	2.16 [1.57, 2.75]	5.59 [4.45, 6.73] ***	7.47 [6.18, 8.78] ***
Data-driven stage IV	1.94 [1.30, 2.58]	3.97 [2.75, 5.17] ***	6.97 [5.61, 8.39] ***
Data-driven stage V	1.61 [1.01, 2.21]	2.95 [1.87, 4.05] ***	6.50 [5.22, 7.78] ***
Temporal meta-ROI	2.39 [1.75, 3.03] ***	5.86 [4.69, 7.09] ***	7.32 [5.94, 8.70] ***
Whole brain meta-ROI	1.99 [1.38, 3.58]	4.13 [2.92, 5.34] ***	7.04 [5.76, 8.30] ***
Q1	5.59 [4.56, 6.62] ***	7.64 [6.48, 8.80] ***	8.54 [7.15, 9.77] ***
Probability-based	5.89 [4.94, 6.84] ***	7.92 [6.71, 9.13] ***	9.20 [7.71, 10.4] ***
Overlap Index	5.14 [4.15, 6.13] ***	6.61 [5.47, 7.75] ***	7.74 [6.36, 9.12] ***
SUSTAIN	5.32 [4.33, 6.31] ***	7.03 [5.91, 8.15] ***	8.15 [6.78, 9.52] ***
Data-driven stage $_{\text{Highest Tau+}}$	6.69 [5.83, 7.55] ***	8.67 [7.49, 9.85] ***	10.74 [9.33, 12.20] ***

© 2023 Leuzy A et al. *JAMA Neurology.*

Aβ indicates amyloid-β; CU, cognitively unimpaired; MCI, mild cognitive impairment; AD, Alzheimer disease. ROI, region of interest; Q1, quartile 1; SUSTAIN, Subtype and Stage Inference; DD, Data Driven. Mean annual change for the DD Stage $_{\text{Highest Tau+}}$ approach is based on Gaussian mixture modelling (GMM) based SUVR cut-offs: data-driven stage I > 1.27; data-driven stage II > 1.39; data-driven stage III > 1.43; data-driven stage IV > 1.26; data-driven stage V > 1.30. Using the highest tau-PET positive Braak stage at baseline instead of the data-driven ROIs (Braak Stage $_{\text{Highest Tau+}}$) resulted in findings similar to those using DD Stage $_{\text{Highest Tau+}}$ (Aβ-positive CU, 6.45 [5.80, 7.62]; Aβ-positive MCI, 8.35 [7.02, 9.68]; AD dementia, 9.86 [8.19, 11.50]) using GMM-based SUVR cut-offs (Braak I > 1.40; Braak II > 1.24; Braak III > 1.36; Braak IV > 1.43; Braak V > 1.28; Braak VI > 1.22). Similar findings were obtained using SUVR cut-offs based on the mean plus 2 standard deviations from amyloid-β negative CU individuals (n=172): data-driven stage I > 1.26; data-driven stage II > 1.44; data-driven stage III > 1.45; data-driven stage IV > 1.33; data-driven stage V > 1.36 (Aβ-positive CU, 6.33 [5.32, 7.96]; Aβ-positive MCI, 8.27 [7.03, 9.51]; AD dementia, 9.48 [7.38, 11.60]); Braak I > 1.42; Braak II > 1.22; Braak III > 1.31; Braak IV > 1.47; Braak V > 1.26; Braak VI > 1.28 (Aβ-positive CU, 6.36 [5.26, 7.46]; Aβ-positive MCI, 8.16 [6.70, 9.62]; AD dementia, 9.86 [7.88, 11.80]).

© 2023 Leuzy A et al. *JAMA Neurology.*

eTable 5. Mean annual change in [^{18}F]RO948 tau PET SUVR

	**Mean annual change [95% CI]**		
**ROI**	**Aβ-positive CU**	**Aβ-positive MCI**	**AD dementia**
Data-driven stage I	0.046 [0.037, 0.055] ***	0.058 [0.038, 0.078] ***	0.046 [0.014, 0.078] ***
Data-driven stage II	0.025 [0.018, 0.032] *	0.096 [0.067, 0.125] ***	0.231 [0.175, 0.287] ***
Data-driven stage III	0.022 [0.016, 0.028]	0.095 [0.067, 0.123] ***	0.222 [0.165, 0.279] ***
Data-driven stage IV	0.018 [0.012, 0.024]	0.040 [0.024, 0.057] ***	0.120 [0.085, 0.155] ***
Data-driven stage V	0.015 [0.009, 0.021]	0.036 [0.022, 0.049] ***	0.106 [0.078, 0.134] ***
Temporal meta-ROI	0.048 [0.022, 0.074]	0.017 [0.080, 0.154] ***	0.237 [0.177, 0.297] ***
Whole brain meta-ROI	0.023 [0.016, 0.037]	0.061 [0.042, 0.080] ***	0.159 [0.114, 0.204] ***
Q1	0.097 [0.077, 0.118] ***	0.120 [0.098, 0.142] ***	0.150 [0.127, 0.174] ***
Probability-based	0.067 [0.055, 0.078] ***	0.137 [0.111, 0.173] ***	0.132 [0.108, 0.155] ***
Overlap Index	0.084 [0.067, 0.102] ***	0.094 [0.075, 0.113] ***	0.113 [0.089, 0.137] ***
SUSTAIN	0.062 [0.048, 0.088] ***	0.131 [0.102, 0.160] ***	0.147 [0.118, 0.176] ***
Data-driven stage $_{\text{Highest Tau+}}$	0.066 [0.058, 0.076] ***	0.145 [0.115, 0.174] ***	0.267 [0.212, 0.322] ***

© 2023 Leuzy A et al. *JAMA Neurology.*

Aβ indicates amyloid-β; CU, cognitively unimpaired; MCI, mild cognitive impairment; AD, Alzheimer disease. ROI, region of interest; Q1, quartile 1; SUSTAIN, Subtype and Stage Inference; DD, Data Driven. Mean annual change for the DD Stage $_{\text{Highest Tau+}}$ approach is based on Gaussian mixture modelling (GMM) based SUVR cut-offs: data-driven stage I > 1.27; data-driven stage II > 1.39; data-driven stage III > 1.43; data-driven stage IV > 1.26; data-driven stage V > 1.30. Using the highest tau-PET positive Braak stage at baseline instead of the data-driven ROIs (Braak Stage $_{\text{Highest Tau+}}$) resulted in findings similar to those using DD Stage $_{\text{Highest Tau+}}$ (Aβ-positive CU, 6.45 [5.80, 7.62]; Aβ-positive MCI, 8.35 [7.02, 9.68]; AD dementia, 9.86 [8.19, 11.50]) using GMM-based SUVR cut-offs (Braak I > 1.40; Braak II > 1.24; Braak III > 1.36; Braak IV > 1.43; Braak V > 1.28; Braak VI > 1.22). Similar findings were obtained using SUVR cut-offs based on the mean plus 2 standard deviations from amyloid-β negative CU individuals (n=172): data-driven stage I > 1.26; data-driven stage II > 1.44; data-driven stage III > 1.45; data-driven stage IV > 1.33; data-driven stage V > 1.36 (Aβ-positive CU, 6.33 [5.32, 7.96]; Aβ-positive MCI, 8.27 [7.03, 9.51]; AD dementia, 9.48 [7.38, 11.60]); Braak I > 1.42; Braak II > 1.22; Braak III > 1.31; Braak IV > 1.47; Braak V > 1.26; Braak VI > 1.28 (Aβ-positive CU, 6.36 [5.26, 7.46]; Aβ-positive MCI, 8.16 [6.70, 9.62]; AD dementia, 9.86 [7.88, 11.80]).

© 2023 Leuzy A et al. *JAMA Neurology.*

eTable 6. Mean annual percent change in [^{18}F]RO948 tau PET SUVR using Braak ROIs

	**Mean annual change [95% CI]**		
**ROI**	**Aβ-positive CU**	**Aβ-positive MCI**	**AD dementia**
Braak I	4.62 [3.38, 5.86] ***	5.14 [3.74, 6.56] ***	3.82 [1.94, 5.70]
Braak II	2.55 [1.54, 3.61] ***	2.53 [1.25, 3.81] ***	1.89 [0.51, 3.19] *
Braak III	2.41 [1.42, 3.42] *	5.98 [4.62, 7.34] ***	7.28 [5.77, 8.79] ***
Braak IV	1.72 [0.95, 2.49]	6.25 [4.75, 7.75] ***	7.97 [6.29, 9.69] ***
Braak V	1.48 [0.68, 2.28]	2.96 [1.80, 1.42] ***	7.07 [5.41, 8.73] ***
Braak VI	1.12 [0.64, 1.77]	1.79 [0.78, 2.80]	4.07 [2.80, 5.34] ***
Braak $_{\text{Highest Tau+}}$	6.91 [6.03, 7.79] ***	9.01 [7.83, 10.20] ***	10.92 [9.68, 12.20] ***

Aβ indicates amyloid-β; CU, cognitively unimpaired; MCI, mild cognitive impairment; AD, Alzheimer disease; ROI, region of interest.

© 2023 Leuzy A et al. *JAMA Neurology.*

eTable 7. Mean annual change in [^{18}F]RO948 tau PET SUVR using Braak ROIs

	**Mean annual change [95% CI]**		
**ROI**	**Aβ-positive CU**	**Aβ-positive MCI**	**AD dementia**
Braak I	0.052 [0.038, 0.067] ***	0.088 [0.060, 0116] ***	0.090 [0.038, 0.142]
Braak II	0.028 [0.017, 0.040] ***	0.033 [0.015, 0.052] ***	0.028 [0.005, 0.051] ***
Braak III	0.035 [0.021, 0.049] *	0.0118 [0.082, 0.154] ***	0.215 [0.160, 0.270] ***
Braak IV	0.023 [0.007, 0.039]	0.129 [0.085, 0.173] ***	0.273 [0.201, 0.345] ***
Braak V	0.022 [0.010, 0.036]	0.058 [0.037, 0.079] ***	0.191 [0.147, 0.235] ***
Braak VI	0.015 [0.007, 0.023]	0.026 [0.013, 0.039]	0.086 [0.059, 0.113] ***
Braak $_{\text{Highest Tau+}}$	0.083 [0.071, 0.096] ***	0.157 [0.128, 0.186] ***	0.305 [0.243, 0.367] ***

Aβ indicates amyloid-β; CU, cognitively unimpaired; MCI, mild cognitive impairment; AD, Alzheimer disease; ROI, region of interest.

© 2023 Leuzy A et al. *JAMA Neurology.*

eTable 8. Distribution of tau PET positivity across data-driven and Braak ROIs using [^{18}F]RO948

		Mean number (SD) of tau-PET positive ROIs	
**Group**	**N**	**Data-driven**	**Braak**
Aβ-positive CU	97	14 (18.49)	15.33 (12.27)
Aβ-positive MCI	77	23.60 (14.17)	31.33 (9.73)
AD dementia	41	27 (10.79)	33.67 (4.18)

Results are based on Gaussian mixture modelling (GMM) based SUVR cut-offs: data-driven stage I > 1.33; data-driven stage II > 1.44; data-driven stage III > 1.51; data-driven stage IV > 1.45; data-driven stage V > 1.41; Braak I > 1.40; Braak II > 1.24; Braak III > 1.36; Braak IV > 1.43; Braak V > 1.28; Braak VI > 1.22)

© 2023 Leuzy A et al. *JAMA Neurology.*

eTable 9. Comparison of mean annual change in [^{18}F]RO948 tau PET SUVR between group-level and individualized ROIs

Comparison		P-values from paired t-test		
**Individualized ROI**	**Group-level ROI**	**Aβ-positive CU**	**Aβ-positive MCI**	**AD dementia**
Q1	Best data-driven ROI	<0.05	<0.05	0.27
	Temporal meta-ROI	<0.05	<0.05	0.18
Probability-based	Best data-driven ROI	<0.05	<0.01	<0.001
	Temporal meta-ROI	<0.01	<0.01	<0.001
Overlap Index	Best data-driven ROI	0.09	0.39	0.63
	Temporal meta-ROI	<0.05	0.42	0.69
SUSTAIN	Best data-driven ROI	<0.05	0.07	0.51
	Temporal meta-ROI	<0.05	0.07	0.48
Data-driven stage $_{\text{Highest Tau+}}$	Best data-driven ROI	<0.001	<0.001	<0.001
	Temporal meta-ROI	<0.001	<0.001	<0.001

Aβ indicates amyloid-β; CU, cognitively unimpaired; MCI, mild cognitive impairment; AD, Alzheimer disease; ROI, region of interest; Q1, quartile 1; SUSTAIN, Subtype and Stage Inference.

© 2023 Leuzy A et al. *JAMA Neurology.*

eTable 10. [^{18}F]RO948-based sample size estimations for group-level and individualized ROIs assuming a 20% intervention effect

	**Required *n* per arm to detect an intervention effect of 20%**		
**Target ROI for assessing tau-PET change**	**Aβ-positive CU**	**Aβ-positive MCI**	**AD dementia**
***Group-level* ROIs**			
Data-driven stage I	411	844	851
Data-driven stage II	749	311	220
Data-driven stage III	866	355	203
Data-driven stage IV	1136	752	235
Data-driven stage V	1327	1113	273
Braak I	311	737	1325
Braak II	459	985	451
Braak III	929	377	299
Braak IV	3065	446	322
Braak V	2893	1168	380
Braak VI	4032	2484	498

© 2023 Leuzy A et al. *JAMA Neurology.*

Temporal meta-ROI	649	328	221
Whole brain meta-ROI	821	670	268
***Individualized* ROIs**			
Q1	315	202	150
Probability-based	196	189	136
Overlap Index	371	276	207
SUSTAIN	338	233	182
Data-driven stage $_{\text{Highest Tau+}}$	181	154	114

Aβ indicates amyloid-β; CU, cognitively unimpaired; MCI, mild cognitive impairment; AD, Alzheimer disease; ROI, region of interest; Q1, quartile 1; SUSTAIN, Subtype and Stage Inference.

© 2023 Leuzy A et al. *JAMA Neurology.*

eTable 11. [^{18}F]RO948-based sample size estimations for group-level and individualized ROIs assuming a 30% intervention effect

	**Required *n* per arm to detect an intervention effect of 30%**		
**Target ROI for assessing tau-PET change**	**Aβ-positive CU**	**Aβ-positive MCI**	**AD dementia**
***Group-level* ROIs**			
Data-driven stage I	183	375	379
Data-driven stage II	333	139	98
Data-driven stage III	385	158	91
Data-driven stage IV	505	335	105
Data-driven stage V	590	495	122
Braak I	139	328	590
Braak II	205	439	201
Braak III	413	168	133
Braak IV	1363	199	143
Braak V	1286	520	169
Braak VI	1793	1104	222

© 2023 Leuzy A et al. *JAMA Neurology.*

Temporal meta-ROI	289	146	99
Whole brain meta-ROI	365	298	120
***Individualized* ROIs**			
Q1	141	90	67
Probability-based	88	84	61
Overlap Index	166	123	92
SUSTAIN	151	104	81
Data-driven stage $_{\text{Highest Tau+}}$	81	69	51

Aβ indicates amyloid-β; CU, cognitively unimpaired; MCI, mild cognitive impairment; AD, Alzheimer disease; ROI, region of interest; Q1, quartile 1; SUSTAIN, Subtype and Stage Inference.

© 2023 Leuzy A et al. *JAMA Neurology.*

eTable 12. [^{18}F]RO948-based sample size estimations for group-level and individualized ROIs assuming a 40% intervention effect

	**Required *n* per arm to detect an intervention effect of 40%**		
**Target ROI for assessing tau-PET change**	**Aβ-positive CU**	**Aβ-positive MCI**	**AD dementia**
***Group-level* ROIs**			
Data-driven stage I	104	212	213
Data-driven stage II	188	78	56
Data-driven stage III	217	89	52
Data-driven stage IV	285	189	59
Data-driven stage V	332	279	69
Braak I	78	185	332
Braak II	116	247	114
Braak III	233	95	75
Braak IV	767	112	81
Braak V	724	293	96
Braak VI	1009	622	125

© 2023 Leuzy A et al. *JAMA Neurology.*

Temporal meta-ROI	163	83	56
Whole brain meta-ROI	206	168	68
***Individualized* ROIs**			
Q1	79	51	38
Probability-based	50	48	35
Overlap Index	94	70	52
SUSTAIN	85	59	46
Data-driven stage $_{\text{Highest Tau+}}$	46	39	29

Aβ indicates amyloid-β; CU, cognitively unimpaired; MCI, mild cognitive impairment; AD, Alzheimer disease; ROI, region of interest; Q1, quartile 1; SUSTAIN, Subtype and Stage Inference.

© 2023 Leuzy A et al. *JAMA Neurology.*

eTable 13. Sensitivity Analysis Comparing Sample Size Reductions Between Tau Extent and Annual Percent Change in SUVR in the Temporal Meta-ROI using [^{18}F]RO948

	**Required *n* per arm to detect an intervention effect of 30%**		
	**Aβ-positive CU**	**Aβ-positive MCI**	**AD dementia**
**Temporal meta-ROI**			
Annual percent change in Tau Extent	352	207	165
Annual percent change in SUVR	289	146	109

Aβ indicates amyloid-β; CU, cognitively unimpaired; MCI, mild cognitive impairment; AD, Alzheimer disease; ROI, region of interest; SUVR, standardized uptake value ratio.

© 2023 Leuzy A et al. *JAMA Neurology.*

eTable 14. Participant characteristics in the [^{18}F]flortaucipir validation sample

	**Aβ-positive CU**	**Aβ-positive MCI**	**AD dementia**
N	137	144	125
Age, y	72.07 (6.73)	71.87 (7.37)	73.07 (9.16)
Sex, M/ F (% F)	80 (58%)	71 (49%)	60 (48%)
Education, y	29.03 (1.17)	27.44 (2.14)	24.02 (1.63)
MMSE score	137	144	125
Tau-PET, scan interval, y	1.71 (0.43)	1.78 (0.39)	1.59 (0.40)

Aβ indicates amyloid-β; CU, cognitively unimpaired; MCI, mild cognitive impairment; AD, Alzheimer disease; MMSE, mini mental state examination; PET, positron emission tomography.

© 2023 Leuzy A et al. *JAMA Neurology.*

eTable 15. Mean annual percent change in [^{18}F]flortaucipir tau PET SUVR

	Mean annual change [95% CI]		
**ROI**	**Aβ-positive CU**	**Aβ-positive MCI**	**AD dementia**
Data-driven stage I	3.28 [2.50, 3.92]	3.50 [2.54, 4.46]	2.94 [2.22, 3.66]
Data-driven stage II	2.12 [1.67, 2.75]	5.41 [4.51, 6.31]	5.43 [4.29, 6.57]
Data-driven stage III	1.83 [1.41, 2.25]	4.82 [3.80, 5.84]	6.08 [4.90, 7.26]
Data-driven stage IV	1.63 [1.11, 2.15]	4.12 [3.13, 5.11]	5.81 [4.61, 7.01]
Data-driven stage V	1.11 [0.55, 1.67]	2.56 [1.84, 3.28]	5.33 [4.33, 6.33]
Temporal meta-ROI	1.58 [1.58, 2.74]	5.16 [4.29, 6.43]	6.14 [4.81, 7.47]
Whole brain meta-ROI	1.60 [1.06, 2.14]	3.87 [3.06, 4.68]	5.69 [4.52, 6.86]
Q1	4.73 [3.95, 5.52]	6.36 [5.26, 7.40]	7.22 [6.26, 8.38]
Probability-based	5.18 [4.36, 6.01]	6.53 [5.80, 7.46]	7.84 [6.76, 9.12]
Overlap Index	4.23 [3.39, 5.07]	5.82 [4.90, 6.74]	6.39 [5.34, 7.66]
SUSTAIN	4.61 [3.72, 5.49]	6.14 [5.19, 7.09]	6.62 [5.80, 7.84]
Data-driven stage $_{\text{Highest Tau+}}$	5.37 [4.66, 6.28]	6.86 [6.05, 7.67]	8.36 [7.58, 9.74]

© 2023 Leuzy A et al. *JAMA Neurology.*

Aβ indicates amyloid-β; CU, cognitively unimpaired; MCI, mild cognitive impairment; AD, Alzheimer disease. ROI, region of interest; Q1, quartile 1; SUSTAIN, Subtype and Stage Inference; DD, Data Driven. Mean annual change for the DD Stage $_{\text{Highest Tau+}}$ approach is based on Gaussian mixture modelling (GMM) based SUVR cut-offs: data-driven stage I > 1.33; data-driven stage II > 1.44; data-driven stage III > 1.51; data-driven stage IV > 1.45; data-driven stage V > 1.41. Using the highest tau-PET positive Braak stage at baseline instead of the data-driven ROIs (Braak Stage $_{\text{Highest Tau+}}$) resulted in findings similar to those using DD Stage $_{\text{Highest Tau+}}$ (Aβ-positive CU, 5.22 [4.42, 4.91]; Aβ-positive MCI, 6.42 [5.54, 7.30]; AD dementia, 8.31 [7.21, 9.43]) using GMM-based SUVR cut-offs (Braak I > 1.35; Braak II > 1.32; Braak III > 1.37; Braak IV > 1.55; Braak V > 1.49; Braak VI > 1.41). Similar findings were obtained using SUVR cut-offs based on the mean plus 2 standard deviations from amyloid-β negative CU individuals (n=184): data-driven stage I > 1.34; data-driven stage II > 1.42; data-driven stage III > 1.43; data-driven stage IV > 1.39; data-driven stage V > 1.38 (Aβ-positive CU, 5.23 [4.26, 6.46]; Aβ-positive MCI, 6.45 [5.49, 7.39]; AD dementia, 8.16 [7.58, 9.54]); Braak I > 1.34; Braak II > 1.40; Braak III > 1.43; Braak IV > 1.47; Braak V > 1.39 ; Braak VI > 1.32 (Aβ-positive CU, 5.17 [4.48, 5.86]; Aβ-positive MCI, 6.38 [5.47, 7.29]; AD dementia, 8.27 [7.72, 9.72]).

© 2023 Leuzy A et al. *JAMA Neurology.*

eTable 16. Mean annual change in [^{18}F]flortaucipir tau PET SUVR

	**Mean annual change [95% CI]**		
**ROI**	**Aβ-positive CU**	**Aβ-positive MCI**	**AD dementia**
Data-driven stage I	0.060 [0.046, 0.074]	0.041 [0.029, 0.053]	0.045 [0.034, 0.060]
Data-driven stage II	0.030 [0.021, 0.039]	0.099 [0.080, 0.118]	0.108 [0.082, 0.134]
Data-driven stage III	0.023 [0.017, 0.029]	0.086 [0.064, 0.108]	0.119 [0.090, 0.148]
Data-driven stage IV	0.022 [0.015, 0.029]	0.070 [0.048, 0.092]	0.100 [0.076, 0.124]
Data-driven stage V	0.014 [0.007, 0.024]	0.034 [0.025, 0.044]	0.085 [0.064, 0.106]
Temporal meta-ROI	0.031 [0.022, 0.040]	0.103 [0.082, 0.124]	0.131 [0.097, 0.164]
Whole brain meta-ROI	0.022 [0.015, 0.032]	0.064 [0.049, 0.080]	0.091 [0.061, 0.121]
Q1	0.072 [0.058, 0.087]	0.087 [0.071, 0.103]	0.096 [0.078, 0.114]
Probability-based	0.082 [0.067, 0.096]	0.096 [0.084, 0.108]	0.120 [0.099, 0.141]
Overlap Index	0.065 [0.052, 0.0784]	0.095 [0.079, 0.110]	0.113 [0.092, 0.134]
SUSTAIN	0.084 [0.067, 0.102]	0.087 [0.075, 0.099]	0.095 [0.082, 0.108]
Data-driven stage $_{\text{Highest Tau+}}$	0.087 [0.0691, 0.098]	0.097 [0.097, 0.128]	0.167 [0.145, 0.189]

© 2023 Leuzy A et al. *JAMA Neurology.*

Aβ indicates amyloid-β; CU, cognitively unimpaired; MCI, mild cognitive impairment; AD, Alzheimer disease. ROI, region of interest; Q1, quartile 1; SUSTAIN, Subtype and Stage Inference; DD, Data Driven. Mean annual change for the DD Stage $_{\text{Highest Tau+}}$ approach is based on Gaussian mixture modelling (GMM) based SUVR cut-offs: data-driven stage I > 1.33; data-driven stage II > 1.44; data-driven stage III > 1.51; data-driven stage IV > 1.45; data-driven stage V > 1.41. Using the highest tau-PET positive Braak stage at baseline instead of the data-driven ROIs (Braak Stage $_{\text{Highest Tau+}}$) resulted in findings similar to those using DD Stage $_{\text{Highest Tau+}}$ (Aβ-positive CU, 6.69 [5.83, 7.55]; Aβ-positive MCI, 8.67 [7.49, 9.85]; AD dementia, 10.74 [9.33, 12.20]) using GMM-based SUVR cut-offs (Braak I > 1.35; Braak II > 1.32; Braak III > 1.37; Braak IV > 1.55; Braak V > 1.49; Braak VI > 1.41). Similar findings were obtained using SUVR cut-offs based on the mean plus 2 standard deviations from amyloid-β negative CU individuals (n=184): data-driven stage I > 1.34; data-driven stage II > 1.42; data-driven stage III > 1.43; data-driven stage IV > 1.39; data-driven stage V > 1.38; Braak I > 1.34; Braak II > 1.40; Braak III > 1.43; Braak IV > 1.47; Braak V > 1.39; Braak VI > 1.32).

© 2023 Leuzy A et al. *JAMA Neurology.*

eTable 17. Mean annual percent change in [^{18}F]flortaucipir tau PET SUVR using Braak ROIs

	**Mean annual change [95% CI]**		
**ROI**	**Aβ-positive CU**	**Aβ-positive MCI**	**AD dementia**
Braak I	3.05 [2.44, 3.66]	3.43 [2.79, 5.07]	2.44 [1.18, 3.70]
Braak II	2.31 [1.64, 2.98]	2.51 [1.80, 3.22]	2.12 [0.93, 3.31]
Braak III	2.71 [2.20, 3.22]	4.51 [4.10, 5.92]	4.07 [3.03, 5.11]
Braak IV	3.12 [2.53, 3.71]	5.84 [4.78, 6.90]	4.60 [3.49, 5.71]
Braak V	0.97 [0.55, 1.39]	3.86 [2.98, 4.74]	3.87 [2.85, 4.91]
Braak VI	0.65 [0.35, 0.95]	2.27 [1.52, 3.02]	2.67 [1.72, 3.62]
Braak $_{\text{Highest Tau+}}$	5.12 [4.57, 5.68]	7.17 [6.33, 8.01]	7.61 [6.45, 8.77]

Aβ indicates amyloid-β; CU, cognitively unimpaired; MCI, mild cognitive impairment; AD, Alzheimer disease; ROI, region of interest.

© 2023 Leuzy A et al. *JAMA Neurology.*

eTable 18. Mean annual change in [^{18}F]flortaucipir tau PET SUVR using Braak ROIs

	Mean annual change [95% CI]		
**ROI**	**Aβ-positive CU**	**Aβ-positive MCI**	**AD dementia**
Braak I	0.041 [0.032, 0.050]	0.066 [0.047, 0.085]	0.038 [0.015, 0.061]
Braak II	0.320 [0.021, 0.041]	0.035 [0.025, 0.047]	0.025 [0.012, 0.039]
Braak III	0.036 [0.028, 0.044]	0.087 [0.070, 0.104]	0.077 [0.055, 0.099]
Braak IV	0.046 [0.036, 0.056]	0.117 [0.093, 0.141]	0.101 [0.071, 0.131]
Braak V	0.012 [0.006, 0.018]	0.065 [0.048, 0.083]	0.071 [0.048, 0.093]
Braak VI	0.007 [0.004, 0.010]	0.030 [0.021, 0.040]	0.039 [0.024, 0.054]
Braak $_{\text{Highest Tau+}}$	0.071 [0.059, 0.079]	0.124 [0.106, 0.140]	0.142 [0.114, 0.169]

Aβ indicates amyloid-β; CU, cognitively unimpaired; MCI, mild cognitive impairment; AD, Alzheimer disease; ROI, region of interest.

© 2023 Leuzy A et al. *JAMA Neurology.*

eTable 19. [^{18}F]Flortaucipir-based sample size estimations for group-level and individualized ROIs assuming a 20% intervention effect

	**Required *n* per arm to detect an intervention effect of 20%**		
**Target ROI for assessing tau-PET change**	**Aβ-positive CU**	**Aβ-positive MCI**	**AD dementia**
***Group-level* ROIs**			
Data-driven stage I	411	844	851
Data-driven stage II	749	311	220
Data-driven stage III	866	355	203
Data-driven stage IV	1136	752	235
Data-driven stage V	1327	1113	273
Braak I	311	737	1325
Braak II	459	985	451
Braak III	929	377	299
Braak IV	3065	446	322
Braak V	2893	1168	380
Braak VI	4032	2484	498

© 2023 Leuzy A et al. *JAMA Neurology.*

Temporal meta-ROI	649	328	221
Whole brain meta-ROI	821	670	268
***Individualized* ROIs**			
Q1	315	202	150
Probability-based	196	189	136
Overlap Index	371	276	207
SUSTAIN	338	233	182
Data-driven stage $_{\text{Highest Tau+}}$	181	154	114

Aβ indicates amyloid-β; CU, cognitively unimpaired; MCI, mild cognitive impairment; AD, Alzheimer disease; ROI, region of interest; Q1, quartile 1; SUSTAIN, Subtype and Stage Inference.

© 2023 Leuzy A et al. *JAMA Neurology.*

eTable 20. [^{18}F]Flortaucipir-based sample size estimations for group-level and individualized ROIs assuming a 30% intervention effect

	**Required *n* per arm to detect an intervention effect of 30%**		
**Target ROI for assessing tau-PET change**	**Aβ-positive CU**	**Aβ-positive MCI**	**AD dementia**
***Group-level* ROIs**			
Data-driven stage I	183	375	379
Data-driven stage II	333	139	98
Data-driven stage III	385	158	91
Data-driven stage IV	505	335	105
Data-driven stage V	590	495	122
Braak I	139	328	590
Braak II	205	439	201
Braak III	413	168	133
Braak IV	1363	199	143
Braak V	1286	520	169
Braak VI	1793	1104	222

© 2023 Leuzy A et al. *JAMA Neurology.*

Temporal meta-ROI	289	146	99
Whole brain meta-ROI	365	298	120
***Individualized* ROIs**			
Q1	141	90	67
Probability-based	88	84	61
Overlap Index	166	123	92
SUSTAIN	151	104	81
Data-driven stage $_{\text{Highest Tau+}}$	81	69	51

Aβ indicates amyloid-β; CU, cognitively unimpaired; MCI, mild cognitive impairment; AD, Alzheimer disease; ROI, region of interest; Q1, quartile 1; SUSTAIN, Subtype and Stage Inference.

© 2023 Leuzy A et al. *JAMA Neurology.*

eTable 21. [^{18}F]Flortaucipir-based sample size estimations for group-level and individualized ROIs assuming a 40% intervention effect

	**Required *n* per arm to detect an intervention effect of 40%**		
**Target ROI for assessing tau-PET change**	**Aβ-positive CU**	**Aβ-positive MCI**	**AD dementia**
***Group-level* ROIs**			
Data-driven stage I	104	212	213
Data-driven stage II	188	78	56
Data-driven stage III	217	89	52
Data-driven stage IV	285	189	59
Data-driven stage V	332	279	69
Braak I	78	185	332
Braak II	116	247	114
Braak III	233	95	75
Braak IV	767	112	81
Braak V	724	293	96
Braak VI	1009	622	125

© 2023 Leuzy A et al. *JAMA Neurology.*

Temporal meta-ROI	163	83	56
Whole brain meta-ROI	206	168	68
***Individualized* ROIs**			
Q1	79	51	38
Probability-based	50	48	35
Overlap Index	94	70	52
SUSTAIN	85	59	46
Data-driven stage $_{\text{Highest Tau+}}$	46	39	29

Aβ indicates amyloid-β; CU, cognitively unimpaired; MCI, mild cognitive impairment; AD, Alzheimer disease; ROI, region of interest; Q1, quartile 1; SUSTAIN, Subtype and Stage Inference.

© 2023 Leuzy A et al. *JAMA Neurology.*

eFigure. Sensitivity analysis for [^{18}F]RO948 using the cerebellum, brain stem and eroded subcortical white matter as a composite reference region

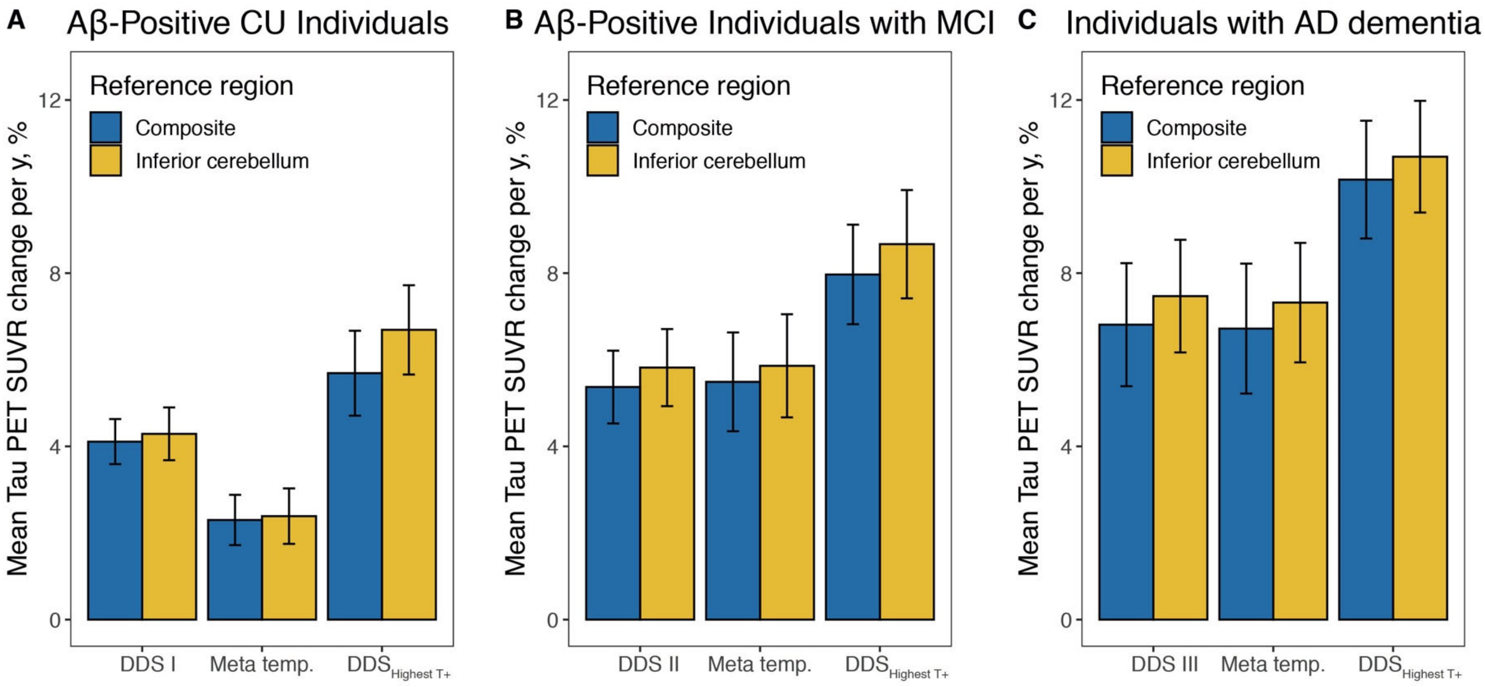

Comparison of mean percent change in [^{18}F]RO948 standardized uptake value ratio per year using the inferior cerebellar cortex and an alternative composite reference region (cerebellum, brain stem and eroded subcortical white matter). Findings are shown for the best performing data-driven stage (DDS), the temporal meta-ROI (Meta temp.) and the highest tau-positive data driven stage (DDS $_{\text{Highest Tau+}}$). Aβ indicates amyloid-β; CU, cognitively unimpaired; MCI, mild cognitive impairment; AD, Alzheimer disease.

© 2023 Leuzy A et al. *JAMA Neurology.*
