# Supplementary material for: Comparison of Group-Level and Individualized Brain Regions for Measuring Change in Longitudinal Tau Positron Emission Tomography in Alzheimer Disease
Source: JAMA Neurol. 2023 May 8;80(6):614–23. doi: 10.1001/jamaneurol.2023.1067 (PMC10167602; doi:10.1001/jamaneurol.2023.1067)
Supplement: Supplement 3. — Data Sharing Statement [file jamaneurol-e231067-s003.pdf]

Data Sharing Statement

Leuzy. Comparison of Group-Level and Individualized Brain Regions for Measuring Change in Longitudinal Tau Positron Emission Tomography in Alzheimer Disease. *JAMA Neurol*. Published May 08, 2023. doi:10.1001/jamaneurol.2023.1067

Data
Data available: No

Additional Information
Explanation for why data not available: Anonymized data will be shared by request from a qualified academic investigator for the sole purpose of replicating procedures and results presented in the article and as long as data transfer is in agreement with EU legislation on the general data protection regulation and decisions by the Ethical Review Board of Sweden and Region Skåne, which should be regulated in a material transfer agreement.
